# Dissecting Tin-Based Activation and Anomerization Pathways in Carbohydrate Chemistry

**DOI:** 10.1021/acsomega.5c10211

**Published:** 2026-01-20

**Authors:** Claudio D. Navo, María J. Moure, Pablo Valverde, Ana Poveda, Gonzalo Jiménez-Osés, Jesús Jiménez-Barbero, Antonio Franconetti

**Affiliations:** † Center for Cooperative Research in Biosciences (CIC bioGUNE), Basque Research and Technology Alliance (BRTA), Derio, Bizkaia 48160, Spain; ‡ Instituto de Investigaciones Químicas—CIC Cartuja, Avda. Americo Vespucio, 42, Sevilla 41092, Spain; § Ikerbasque, Basque Foundation for Science, Bilbao 48009, Spain; ∥ Department of Organic & Inorganic Chemistry, Faculty of Science and Technology, University of the Basque Country, EHU-UPV, Leioa, Bizkaia 48940, Spain; ⊥ Centro de Investigacion Biomedica En Red de Enfermedades Respiratorias, Madrid 28029, Spain; # Departament de Química, Universitat Autònoma de Barcelona, Cerdanyola del Vallès 08193, Spain

## Abstract

Tin-based Lewis acids are widely used in glycosylation chemistry, but their precise mechanistic role is still not fully understood. In this study, we combine ^119^Sn NMR spectroscopy and density functional theory (DFT) calculations to investigate how Sn­(IV) promoters interact with glycosyl donors and influence anomerization. Our results indicate that glycosyl oxocarbenium ions can form stable ion pairs with tin-based counterions, underscoring the relevance of these species in glycosylation processes. The agreement between experimental and computed ^119^Sn chemical shifts provides structural insights into the coordination environment of the tin species. For anomerization, DFT energy profiles support a mechanism involving endocyclic C–O bond cleavage, rotation, and subsequent ring closure. These findings refine our understanding of tin-mediated transformations and offer a framework for rational design of Lewis acid promoters in glycochemistry.

## Graphical abstract

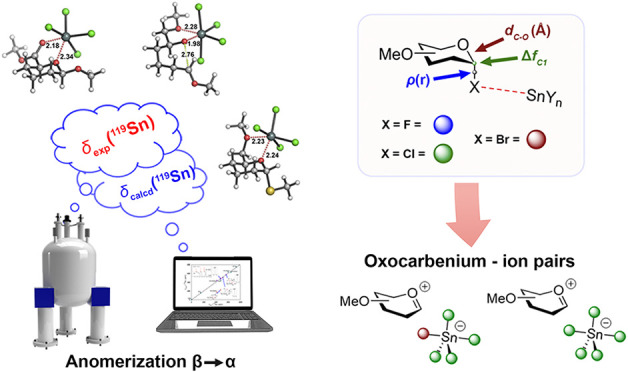

## Introduction

Carbohydrates are essential molecules in everyday life and are among the most stereochemically complex structures in nature. Typically, five chiral centers account for their multiple configurations, while the pyranose ring confers dynamic conformational behavior. Beyond the monosaccharide level, carbohydrate-active enzymes (CAZymes) enable their exquisite assembly,^1^ affording branched structures known as glycans. Variations in glycan sequence, length, and connectivity generate vast structural diversity, expanding their involvement in numerous biological processes closely linked to health and disease.^2^ As a general perspective, glycans are commonly attached to proteins (*O*- or *N*-glycans) or lipids, forming glycoconjugates. The interaction of these glycoconjugates with proteins triggers diverse biological responses, underpinning cell–cell communication and key host–pathogen interactions including molecular recognition in SARS-CoV-2 infection and signaling in cancer and autoimmune disorders.^3–6^

One of the central aspects of glycochemistry is the formation of *O*-glycosidic bonds (C–O–C) through glycosylation reactions, which provide access to complex glycans and glycoconjugates.^7^ This field continues to grow, driven by new methodologies and applications.^8^ Each position within the carbohydrate backbone can be modified; however, glycosylation specifically targets anomeric carbon (C1). Modification at this position generates two possible isomers, α and β anomers. Mechanistically, these reactions span the continuum between classical S_N_1/S_N_1-like and S_N_2/S_N_2-like pathways^9^ and require the assembly of an electrophilic glycosyl donor and a nucleophilic acceptor (Scheme 1). In S_N_1-like processes, coupling proceeds through a short-lived glycosyl oxocarbenium ion.^10^ The nature of the substituent at the anomeric position, usually a leaving group (LG, Scheme 1), strongly influences the donor reactivity. Additional factors such as temperature, concentration, solvent, and particularly the presence of an activator (or promoter) also play a critical role.

**Scheme 1:**
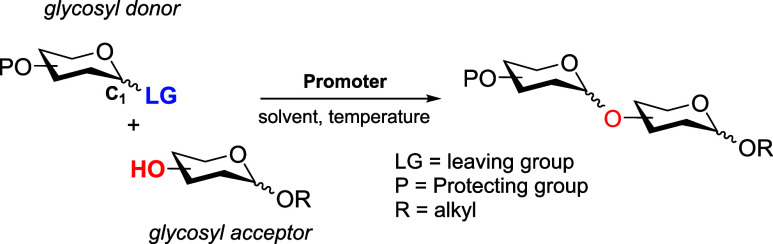
General Overview of Glycosylation Reactions

The first step in glycosylation involves the interaction between the promoter and the glycosyl donor. These species are typically heavy metal salts or Lewis acids, which coordinate to the leaving group.^11^ In most cases, stoichiometric amounts of promoter are required, distinguishing their role from that of catalysts in other metal-mediated glycosylations.^12,13^ Classical promoters include Ag­(I) salts and Lewis acids based on Hg­(II), Sn­(II), or Sn­(IV).^14,15^ Among these, tin stands out over boron, aluminum, and titanium due to its monomeric nature, high solubility in organic solvents, and ease of handling.^16^

Tin-based Lewis acids have also been widely exploited for selective transformations in organic and organometallic chemistry. In particular, SnCl_4_ combines a mild Lewis acidity with remarkable chelation properties. These properties have been extensively studied by NMR, as three tin isotopes possess nonzero spin (1/2) and similar magnetic moments. Among them, ^119^Sn is the most widely used due to its higher natural abundance and gyromagnetic ratio.^17^ Tin nuclei exhibit chemical shifts over an exceptionally broad range (>1800 ppm), and their sensitivity to electronic perturbations makes ^119^Sn NMR a powerful tool for probing structures and elucidating tin species involved in catalytic mechanisms.^18^

The aim of this article is to provide a fresh perspective on tin-based promoters and their role in carbohydrate chemistry. To this end, representative examples have been revisited using a combination of computational methods and NMR spectroscopy (^1^H, ^13^C, and ^119^Sn), offering new insights into current mechanistic understanding. Two main aspects are addressed: (a) the generation of glycosyl cations and (b) Sn-mediated anomerization reactions.

## Results and Discussion

### Generation of Glycosyl Cations: Tin-Based Ion Pairs

Glycosyl halides (**1**, *X* = Cl; **2**, *X* = Br, Scheme 2) were introduced by Koenigs and Knorr as glycosyl donors more than one century ago and have been extensively revisited in recent years.^19^ Lewis acids based on Sn­(IV) species are more frequent than Sn­(II) species in catalysis;^20^ however, glycosylation of “disarmed” glycosyl halides (bearing acetyl protecting groups) has been reported using Sn­(OTf)_2_ as promoter but not Sn­(IV) species, usually in the presence of acid and water scavengers.^21^ A particular case is the SnCl_2_–AgClO_4_ system acting as a fluorophilic activator of glycosyl fluorides (**3**).^22^ Traditionally, Sn­(IV)-promoted glycosylations (e.g., SnCl_4_) required an acetate as the leaving group at the anomeric position.^23,24^ Notably, some recent examples accomplished the glycosylation of halide donors with Lewis acid catalysts, for instance, FeCl_3_ and also SnCl_4_ under catalytic conditions.

**Scheme 2:**
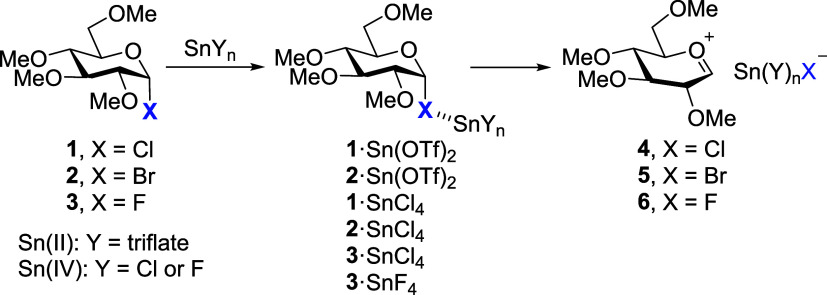
Sn-Promoted Glycosylation of Glycosyl Halides (**1–3**), Their Adducts, and Oxocarbenium–Tin-Based Ion Pairs (**4–6**)

The proposed activation of halide donors involves the initial interaction between the promoter and the leaving group (Scheme 2). The resulting five-coordinated tin complex undergoes promoter-assisted cleavage to form a glycosyl oxocarbenium ion (**4–6**), which is subsequently trapped by the glycosyl acceptor. To analyze this first activation step, we evaluated the interaction between glycosyl halides (**1–3**) and different Sn­(II) and Sn­(IV) promoters by DFT at PCM­(CH_2_Cl_2_)/ωB97X-D/def2-SVP (LANL2DZ for Sn atoms) level. In all models, the electronegative halide occupied the axial position (α-anomer), as expected from the anomeric effect.^25^ Per-*O*-methylated compounds were employed as simplified models for “armed” carbohydrates.

Previous studies have reported that glycosyl halides can be activated through halogen bonding,^26^ a noncovalent interaction based on the presence of a positive electrostatic region (σ-hole) opposite to the C–X bond. This concept is closely related to tetrel bonding, a σ-hole interaction in which group-14 elements act as Lewis acids.^27^ In this context, Sn­(IV) derivatives exhibit a pronounced σ-hole opposite the Sn–X bond, enabling directional interactions with donors containing lone pairs, such as the halogen of the glycosyl donor. The molecular electrostatic potential (MEP, 0.001 au isosurface, see SI) associated with this σ-hole is +42.0 kcal·mol^–1^ for SnCl_4_, + 75.3 kcal·mol^–1^ for SnF_4_, and +60.9 kcal·mol^–1^ for Sn­(OTf)_2_. These values emphasize the ability of Sn­(IV) species to engage in σ-hole interactions, supporting their role in glycosyl halide activation (**1**–**3**). To quantify how these electrostatic features translate into binding strength, we computed interaction energies for the corresponding complexes (in kcal mol^–1^), which follow the trend: **1**·SnCl_4_ (−6.57) ≈ **2**·SnCl_4_ (−6.59) < **3**·SnCl_4_ (−7.3) < **1**·Sn­(OTf)_2_ (−14.1) < **2**·Sn­(OTf)_2_ (−14.3) < **3**·SnF_4_ (−19.4), highlighting the superior activation ability of SnF_4_ and Sn­(OTf)_2_ compared to SnCl_4_. Complexes with SnCl_4_ show weak intermolecular interactions (from −6.6 to −7.3 kcal·mol^–1^) involving halide···Sn contacts, whereas Sn­(OTf)_2_ and SnF_4_ afford larger interaction energies (−14.1 to −19.4 kcal·mol^–1^) and a measurable lengthening of the C–X bond, consistent with their higher ability to activate glycosyl halide donors.

To gain deeper insights into these interactions, we analyzed the electron density within the framework of Bader’s Atoms in Molecules (AIM) theory,^28^ identifying bond critical points and bond paths connecting the σ-hole at Sn with the halide. The activation ability of the Sn-based species was evaluated using three descriptors: (a) *ρ*(r) at the bond critical point of the C–X bond, (b) the equilibrium C–O distance (*d*_C–O_), and (c) the condensed dual descriptor, Δ*f*_C1_ (Figure 1). This condensed dual descriptor is a site-specific reactivity index derived from conceptual DFT, defined as the difference between the nucleophilic and electrophilic Fukui functions (Δ*f* = *f* ^+^–*f* ^–^).^29^ Positive values indicate regions more prone to nucleophilic attack, while negative values correspond to sites susceptible to electrophilic attack. The topological analysis of the C–X bond reveals a progressive decrease of *ρ*(r) upon interaction with different promoters (Figure S1a). Simultaneously, shortening of the C–O bond is observed in all cases. This behavior is most pronounced for glycosyl fluorides (**3**·SnCl_4_ and **3**·SnF_4_). Altogether, these features indicate the initial stage toward glycosyl oxocarbenium formation. To assess the electronic consequences of activation, Fukui functions^30^ and Δ*f*_C1_ (from Hirshfeld charges) were computed to highlight the sites susceptible to nucleophilic attack, focusing on the C1 anomeric carbon (Figure S1b). The results indicate that nucleophilic substitution is initially feasible for glycosyl halides (Δ*f*_C1_ > 0), becomes attenuated upon promoter coordination, and is subsequently restored and even enhanced after oxocarbenium ion formation. The next step for these “activated” species is the departure of the halide group, now coordinated to Sn, which precedes the generation of glycosyl oxocarbenium ions. This cation displays a short C1–O5 distance (ca. 1.25 Å)^31^ and a torsion angle close to zero. The introduction of a positive charge has a pronounced effect on the ring conformation, adopting, for instance, the half-chair ^4^ *H*_3_ conformation in the case of Glc. Participation of a neighboring group such as AcO or BzO at the C2 position leads to the formation of dioxolenium ions instead of glycosyl cations.

**Figure 1:**
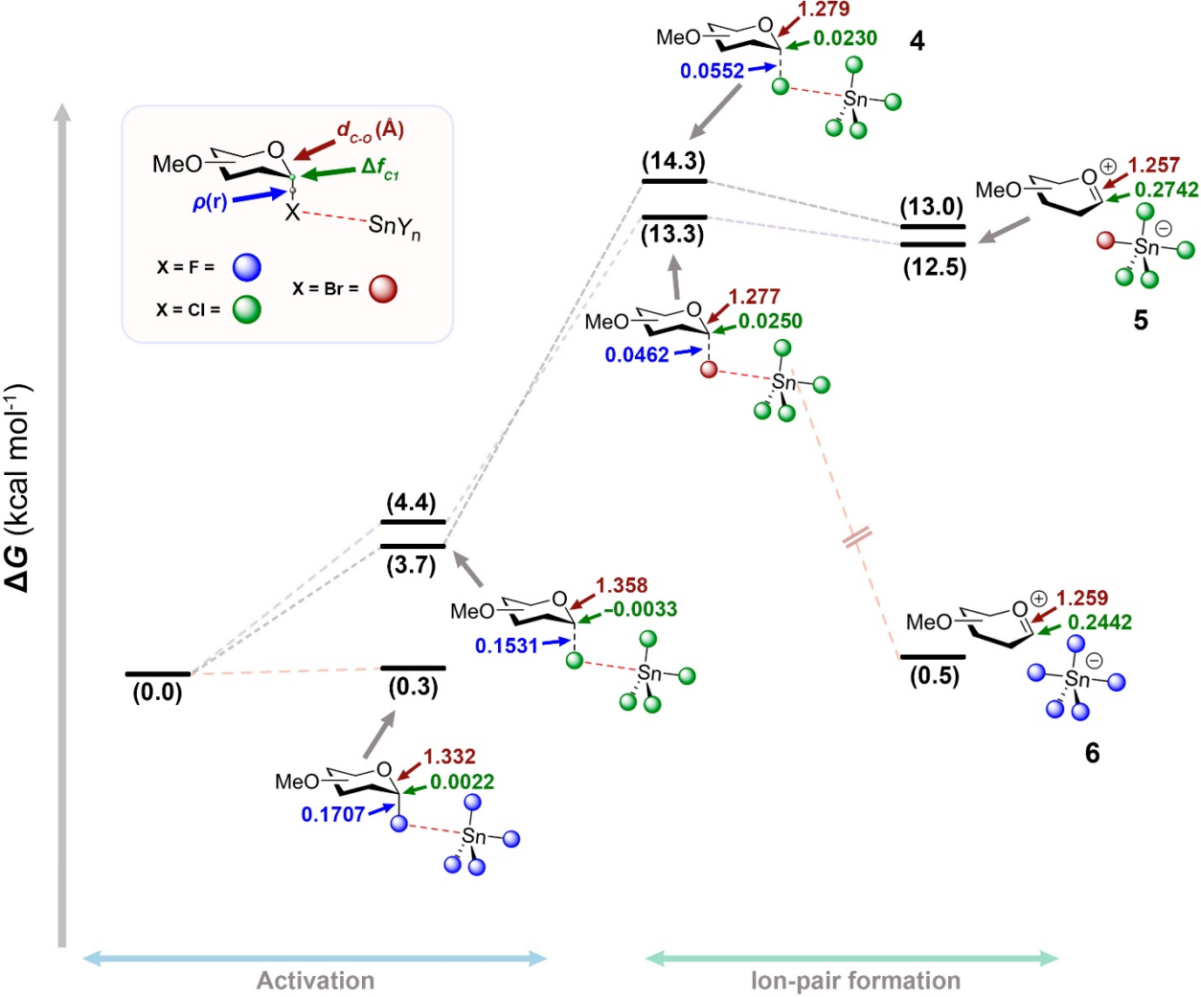
Energy profile (Δ*G*, kcal·mol^–1^) for glycosyl activation and ion-pair formation with Sn-based promoters including key topological and reactive descriptors (*ρ*, *d*_C–O_, and Δ*f*_C1_). Color code for descriptors: density at bond critical point, *ρ*, in blue; and distances are depicted in red and Δ*f*_C1_ in green.

Crich and co-workers highlighted the critical role of counterions in glycosyl cation stability and reactivity.^32^ In this context, we examined highly coordinated tin anions such as SnCl_5_^–^ (complex **4**), SnCl_4_Br^–^ (complex **5**), and SnF_5_^–^ (complex **6**), which have been experimentally and computationally detected and shown to stabilize cationic species.^33^ Contact ion pairs (CIP) between oxocarbenium- and Sn-based anions were identified in all cases. Complexes **4** and **5** display a Δ*G* value of 13.0 and 12.5 kcal·mol^–1^, respectively, indicating weak association, whereas formation of **6** (SnF_5_^–^) is significantly more stable (Δ*G* = 0.5 kcal·mol^–1^). The transition state between **3**·SnF_4_ and **6**·SnF_5_^–^ was not located, suggesting an endergonic reaction consistent with the poor leaving ability of fluorine.

Notably, Δ*f*_C1_ values for **4**–**6** remain comparable to those of the naked oxocarbenium cation (**7**), indicating a similar reactivity at C1. No stable ion pairs were located for Sn­(OTf)_2_-derived species; instead, our computations, consistent with previous experimental observations, suggest that triflate involvement leads to glycosyl triflate intermediates.^34^

The S_N_2 pathway was explored for glycosyl chloride **1** (Δ*f*_C1_ = 0.1416). The calculated barrier for the S_N_2 transition state (Δ*G* ^‡^ = 39.5 kcal·mol^–1^) is significantly higher than that for oxocarbenium formation (Figure S2), supporting the preferential S_N_1 pathway for these glycosyl halides. In addition, the **1**·SnCl_4_ complex is unlikely to follow the S_N_2 pathway (Δ*f*_C1_ = −0.0033) in the presence of MeOH, which promotes the formation of ion-pair **4** instead.

Having established the behavior of halide donors, we turned our attention to per-*O*-acetylated sugars (**8**, Scheme 3) that represent another important class of glycosyl donors. A major challenge in their use is achieving regio- and stereoselective glycosylation. Despite significant progress, current strategies remain limited. Conventional promoters such as AgOTf and Hg­(CN)_2_ have been applied to these donors, and activation with Lewis acids (*e.g*., BF_3_·Et_2_O, TMSOTf, CuOTf, Cu­(OTf)_2_, ZnBr_2_, ZnI_2_, ZnCl_2_) typically affords 1,2-*trans*-glycosides through anchimeric assistance of the C2 acetyl group.^35,36^

**Scheme 3:**
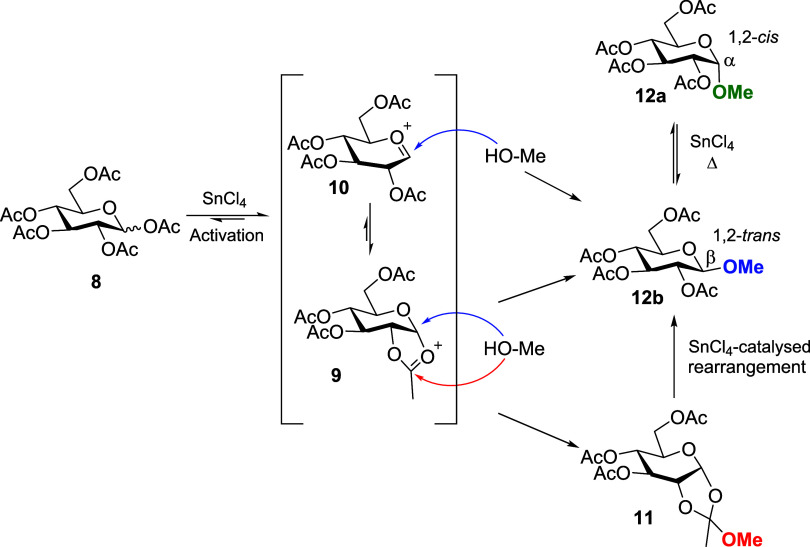
Possible Reaction Pathways for SnCl_4_-Promoted Glycosylation of Per-*O*-acetylated Glycosyl Donors

Upon activation, the glycosyl cation (or dioxolenium ion) can react with a nucleophile to form the 1,2-*trans* glycoside (**12β**) or the 1,2-orthoester (**11**), which may rearrange under acidic conditions to give **12β** (Scheme 3). To probe the stability of these intermediates, we performed quantum mechanical calculations at the ωB97X-D/def2-QZVPPD//ωB97X-D/def2-SVP level (LANL2DZ for Sn) in CH_2_Cl_2_. Dioxolenium **9** was more stable than oxocarbenium **10** (ΔΔ*G* = −2.1 kcal·mol^–1^), but this trend reversed when the counterion was included (ΔΔ*G* = 0.8 kcal·mol^–1^).

Based on the coordination preference of SnCl_4_ (Figure S3), we considered SnCl_4_Ac^–^, formed by bidentate coordination of the leaving acetate to SnCl_4_, which adopts an octahedral geometry. The naked per-*O*-acetylated glycosyl cation (**10**) shows a Δ*f*_C1_ of 0.303, higher than that of the per-*O*-methylated analog (Δ*f*_C1_ = 0.289). Notably, dioxolenium ions exhibit a very low susceptibility to nucleophilic attack (0.0260 and 0.0217 for **9** and **9**·SnCl_4_Ac, respectively) compared to glycosyl cations (**10** and **10**·SnCl_4_Ac). These findings highlight the greater relevance of oxocarbenium-based ion pairs over both naked oxocarbenium and dioxolenium species.

### Tin-Based Anomerization Reactions

Anomerization refers to the interconversion of the absolute configuration at the anomeric carbon in a cyclic carbohydrate. In the case of reducing carbohydrates, this process is also termed mutarotation and occurs naturally in solution via an open-chain intermediate upon cleavage of the endocyclic C–O bond. As a reversible process, it leads to a mixture of both anomers that eventually reaches equilibrium. The ratio of anomers is specific for each carbohydrate and is governed by several factors, such as solvation of the anomeric hydroxyl group, the anomeric effect, 1,3-diaxial interactions, hydrogen bonding, or dipolar repulsion. Conversely, this process is precluded for glycosides, where the anomeric hydroxyl group has been replaced by an ether. Therefore, glycosides are much more stable against anomerization in aqueous solution than their corresponding unsubstituted counterparts.

Although certain substituents such as halides,^37^ dinitrosalicylic acid derivatives,^38^ or triflates^34^ have shown the ability to undergo in situ anomerization in polar solvents, most of these cases require the presence of either a Brønsted or a Lewis acid.^39^ Particularly, Lewis acid-catalyzed anomerization has attracted considerable interest for the stereoselective synthesis of glycosylated derivatives. This process is also modulated by the nature and concentration of the Lewis acid, the carbohydrate protecting groups, and the temperature. In this context, TiCl_4_ ^40^ and BF_3_·Et_2_O^41^ have been extensively employed for catalyzing anomerization reactions.

Sn­(IV) reagents have also shown catalytic activity toward carbohydrate anomerization, albeit to a lesser extent.^42–44^ The mechanism of the Sn­(IV)-catalyzed anomerization reaction has been extensively studied for a number of glycosides.^44^ Experimental outcomes suggest that the reaction proceeds through cleavage of the C1–O5 bond stabilized by chelation with Sn species (Scheme 4a). Nonetheless, an alternative exocyclic cleavage to form an oxocarbenium ion could not be discarded (Scheme 4b).

**Scheme 4:**
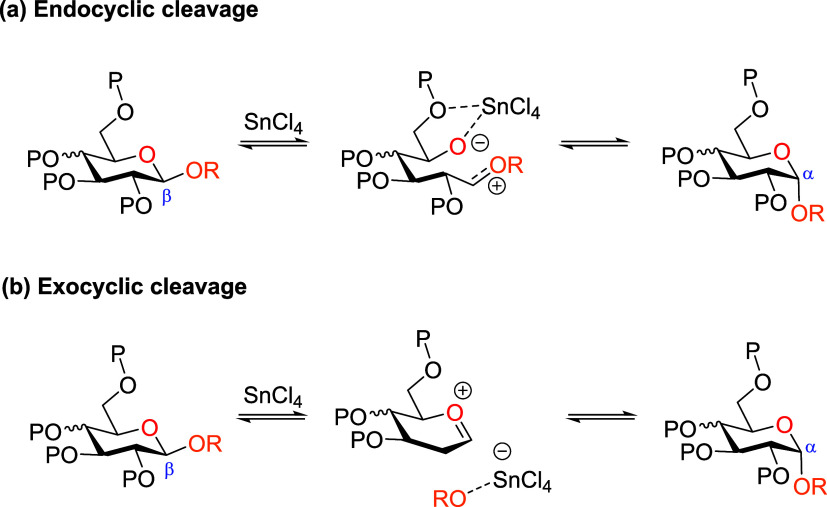
Possible Mechanism of Sn-Promoted *O*-glycoside Anomerization Reaction: (a) Endocyclic and (b) Exocyclic Cleavage^a^

a P: protecting group.

To shed more light on the mechanism of the Sn-catalyzed anomerization reaction, we performed different NMR experiments and a detailed DFT study. First, methyl 2,3,4,6-tetra-*O*-acetyl-β-d-glucopyranoside (**12β**) was synthesized using the standard acetylation procedure with Ac_2_O and pyridine/DMAP in excellent yield (95%, see Supporting Information). Then, the anomerization reaction was carried out in an NMR tube (CDCl_3_ as solvent) following a procedure similar to that described by Murphy and co-workers.^44^ The reaction leading to the α-anomer was monitored by ^1^H, ^13^C, and ^119^Sn NMR.

The δ­(^119^Sn) of SnCl_4_ was −149 ppm (in CDCl_3_). The coordination geometries were further analyzed by the δ­(^119^Sn) values. For this purpose, DFT-calculated chemical shifts were compared with an experimental set of references: SnCl_4_, SnCl_5_^–^, SnCl_4_(Et_2_O)_2_, SnCl_4_(H_2_O)_2_, and SnCl_6_^2–^ (Figure 2), obtaining an excellent correlation (*r* ^2^ = 0.9929) consistent with previous studies on ^119^Sn NMR chemical shift calculations.^45^ Then, ethyl acetate (EtOAc) was employed as a testing compound for our computational methodology. A good theoretical prediction was obtained by coordination through the carbonyl group (−416 vs. −442 ppm). On the other hand, coordination through the EtO moiety (EtOAc, Figure 1) afforded a mismatch with the experimental value (−416 ppm). The experimental scenario proved to be more complex due to the different equilibria in solution. A representative case is the coordination with Et_2_O and how the equilibrium is displaced by changing the temperature (see SI).

**Figure 2:**
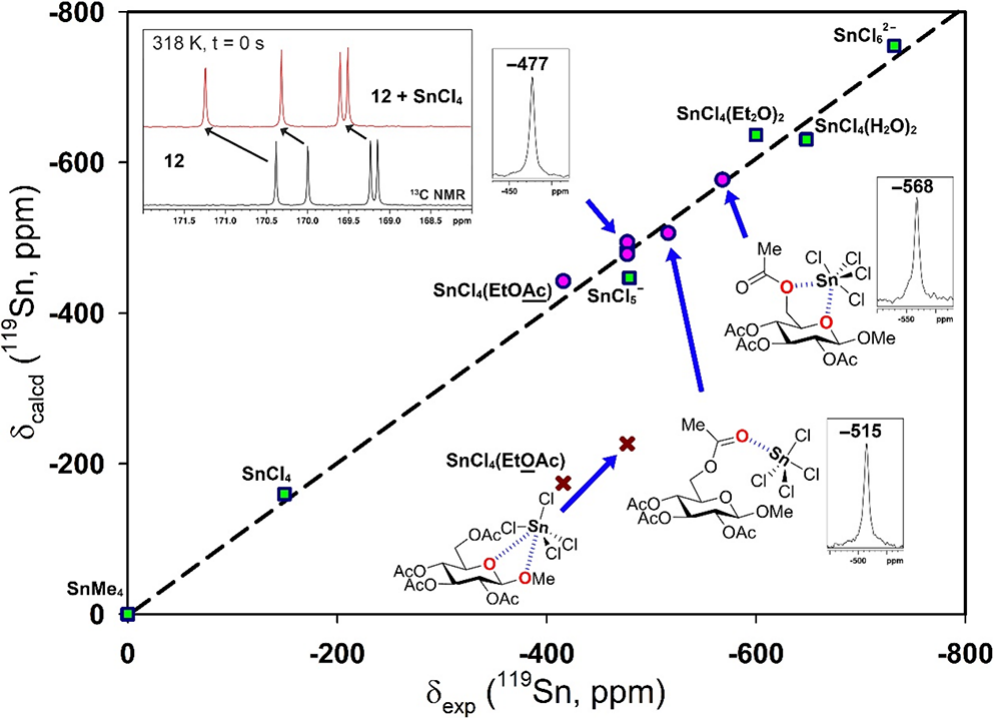
Correlation (in green squares) between experimental and calculated ^119^Sn chemical shifts (in ppm). Circles (pink color) correspond to unknown coordination of SnCl_4_ to EtOAc and compound **12**. Insets show experimental ^119^Sn NMR spectra for each detected species. Crosses (red color) are coordination modes that did not correlate and were finally discarded. Changes on the δ­(^13^C) of carbonyl groups (black spectrum) upon addition of SnCl_4_ (red spectrum) are also shown.

Upon the addition of SnCl_4_ to compound **12β**, an important upfield shift was observed (δ = −477 ppm). This suggests a change in the coordination sphere due to complexation with acetyl groups, forming a pentacoordinated Sn species. In particular, acetyl groups exhibited noticeable changes in their chemical shifts (Table S1), the most significant being the C=O attached to the C6 carbon (Δδ = 1.6 ppm). Coordination with other acetyl groups (C2 and C4) produced similar chemical shift variations (from 0.5 to 0.9 ppm). Theoretical δ­(^119^Sn) values for these coordinated species are consistent with −477 ppm (half-width = 909 Hz). In contrast, the ^1^H NMR spectrum remained unchanged at this initial point (*t* = 0 s). Subsequent ^1^H NMR experiments showed an almost negligible decrease in the signal intensity of all protons until new signals for the α-anomer were detected. Periodically, ^13^C NMR experiments were performed (from 195 to 245 ppm) to detect the corresponding transformation to the oxocarbenium ion (C1 = 228.5 ppm).^46^ In all attempts, the ^13^C NMR spectrum did not show any signal. Over the course of the reaction, the transient ^119^Sn signal at −477 ppm evolved toward a downfield shift (−515 ppm, Figure 2). This signal corresponds to coordination to the *gt* rotamer, adopting a trigonal bipyramidal geometry. Finally, a five-membered chelate ring between SnCl_4_ and the *gg* rotamer of compound **12β** was also observed (−568 ppm, mainly at 298 K). This chemical shift is consistent with a hexacoordinated species involving both O5 and O6.

DFT calculations were applied using different glycoside models for evaluating the influence of the group at the C5 position on both the activation barrier of the process and the relative stability of both anomers (Figure 3 and Table S2). Of note, we were able to find transition structures (TSs) only for the endocyclic cleavage mechanism (Scheme 4a) corresponding to a concerted process that involves breaking of the endocyclic C1–O5 bond and concomitant rotation of the C1–C2 bond (Figure 3b). The coordination of the Sn atom to the endocyclic oxygen is pivotal for stabilizing the negative charge developing throughout the process. The exocyclic cleavage was also evaluated by scanning the potential energy along the breaking C1–O1 bond, although it displayed an uphill profile in all cases without detecting any transition structure. The lack of chelating substituents in model **I-β** (Figure 3a) is significantly detrimental for the calculated activation barrier of the anomerization process (Δ*G* ^‡^ = 24.4 kcal mol^–1^) as a result of the rehybridization of the Sn atom from an octahedral geometry in the equatorial β anomer to a trigonal bipyramidal geometry at the TS. This effect is also observed in the relative stability of the axial α anomer (Δ*G*_α_ = 0.9 kcal mol^–1^), in which the Sn atom also preferably adopts a trigonal bipyramidal geometry. This latter observation follows the experimental trend of reduced axial anomer preference for glycosides lacking substituents at C5.^47^

**Figure 3:**
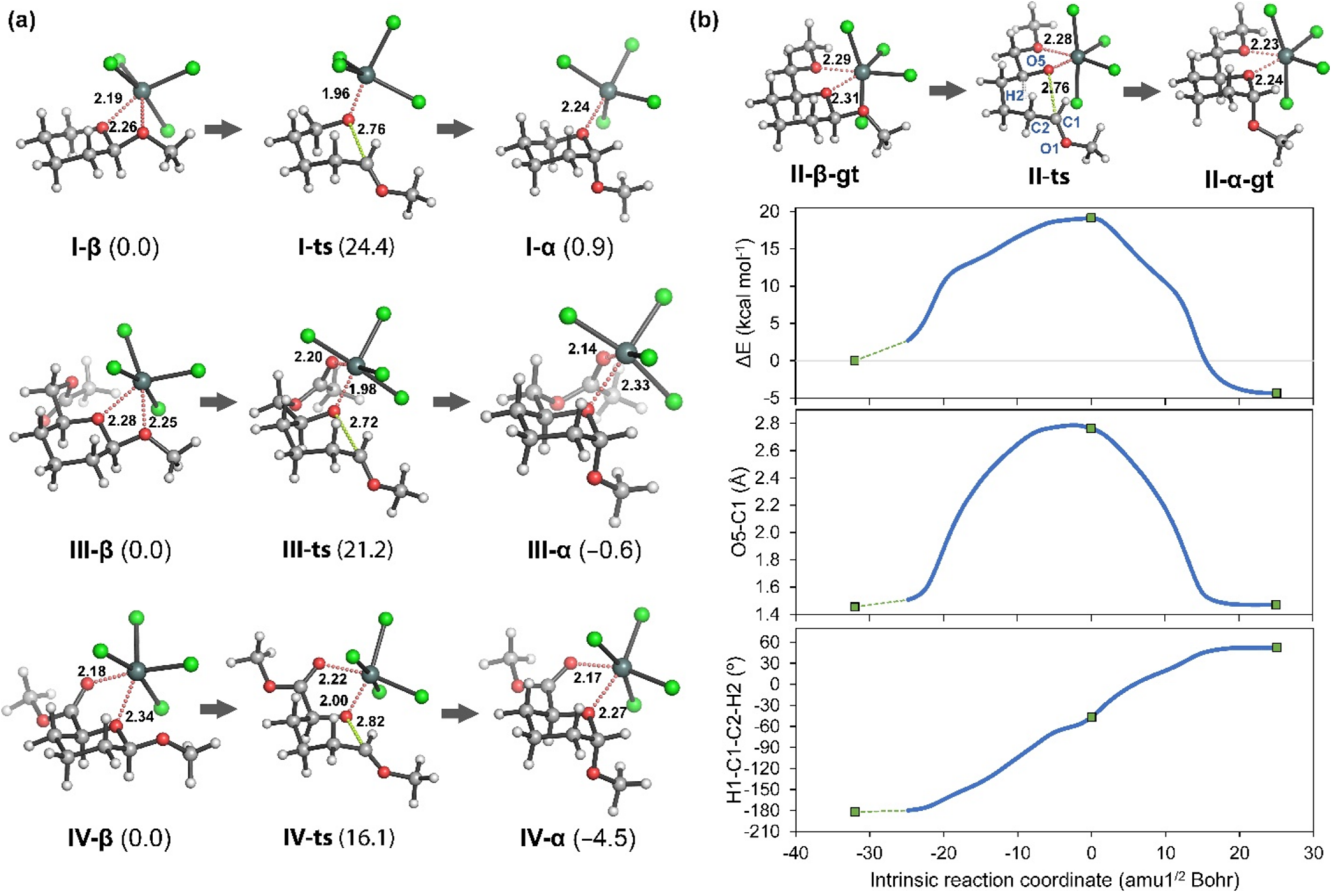
(a) Minimum energy structures and relative stabilities for the transition states and both anomers for the anomerization process of glycoside models **I**, **III**, and **IV**. Relative free Gibbs energies (Δ*G* and Δ*G* ^‡^, given in kcal mol^–1^ in parentheses) were calculated at PCM­(CH_2_Cl_2_)/ωB97X-D/def2-QZVPPD//ωB97X-D/def2-SVP (LanL2DZ for Sn atoms). Distances are given in Å. (b) Geometries for stationary points, intrinsic reaction coordinate (IRC), and variation of the endocyclic O5–C1 bond and the H1–C1–C2–H2 dihedral angle for the anomerization reaction of glycoside model **II**. IRC was calculated with PCM­(CH_2_Cl_2_)/ωB97X-D/def2-SVP (LanL2DZ for Sn atoms) from the minimum energy transition structure (**II**-ts). The methoxymethyl group at C5 is maintained in a gauche–trans (*gt*) conformation in this example.

However, Murphy et al. proved that chelation of SnCl_4_ to a substituent at C5 is not necessary for anomerization to occur, with reaction rates comparable to those found for “disarmed” gluco- and galactopyranosides.

Probably, the absence of substituents at other positions (C2–C4) of our carbohydrate models precludes the formation of stabilizing chelates and causes tin to adopt a different nonoctahedral coordination geometry. On the other hand, the presence of an ether group at C5 (**II-α**, Figure 3b) resulted in a lower activation barrier and a higher relative stability of the α anomer (Δ*G* ^‡^ = 15.0 kcal mol^–1^; Δ*G*_α_ = −4.7 kcal mol^–1^) in comparison to having an acetate group (**III-β**; Δ*G* ^‡^ = 21.2 kcal mol^–1^; Δ*G*_α_ = −0.6 kcal mol^–1^), in agreement with experimental results obtained for protected glycosides. This difference is caused by a change in the coordination environment of the tin atom, which is initially coordinated to both endocyclic and anomeric oxygen atoms in the β anomer and then coordinates to the carbonyl group of the acetate moiety forming a seven membered ring on the TS and the α anomer. As observed in the small models, the interaction of Sn with both a carbonyl and an ether groups is less favorable than with two ether groups, hence increasing the relative energy of the transition structure and the α anomer.

On the other hand, the calculated activation barrier and the α anomer relative energy for the ester derivative (Δ*G* ^‡^ = 16.1 kcal mol^–1^; Δ*G*_α_ = −4.5 kcal mol^–1^) were similar to those found for the ether derivative, matching the experimental outcome reported for protected glucuronic acid derivatives. Free glucuronic acid derivatives exhibited much faster anomerization kinetics than the protected glycosides. However, the calculated activation barrier for this derivative is slightly higher (Figure S4, **V-β**, and Δ*G* ^‡^ = 15.5 kcal mol^–1^) than that of **II-TS**. Finally, the thioglycoside model (**VI-β**, Figure S4) showed a significantly lower activation barrier (Δ*G* ^‡^ = 15.7 kcal mol^–1^) for the anomerization reaction than the analogous oxygen-containing model (Figure 3b), due to the better ability of the sulfur atom to stabilize the partial positive charge upon endocyclic cleavage. This is consistent with the enhanced anomerization rates reported; however, the α adduct was calculated to be much more stable than the β adduct in comparison to the *O*-glycosides. This observation differs from the experimental observation, in which a lower ratio of α anomer is observed upon equilibrium, and can be attributed to a distortion of the anomeric effect due to coordination of O5 to Sn.

## Conclusions

This study provides a detailed view of how tin-based Lewis acids influence the carbohydrate reactivity at different stages. Sn­(IV) species efficiently activate glycosyl fluorides in glycosylation reactions, whereas DFT calculations reveal the limited activation of other glycosyl halides (Cl and Br). Their interaction with Sn­(IV) is weak, and key descriptors (ρ, C–O bond length, and Δ*f*_C1_) indicate that the formation of glycosyl oxocarbenium ions is diffuculted under these conditions. Although glycosyl halides inherently exhibit a nucleophilic substitution capability (Δ*f*_C1_ > 0), coordination to Sn­(IV) further reduces this reactivity.

A different scenario emerges for glycosyl donors bearing an acetyl group at the anomeric position. In these systems, the formation of oxocarbenium–Sn-based ion pairs is thermodynamically more favorable than the generation of dioxolenium species, underscoring the critical role of the leaving group in dictating activation pathways.

The combination of experimental ^119^Sn NMR data and theoretical calculations allowed us to identify distinct coordination geometries for the key intermediates. SnCl_4_ preferentially coordinates to the acetyl group at C6, forming a pentacoordinated intermediate that dynamically evolves toward a chelated structure involving O5 and O6, consistent with a hexacoordinated SnCl_4_ derivative. This complexation lowers the energy barrier for the transition state, supporting a concerted mechanism involving simultaneous C1–O5 bond cleavage and C1–C2 bond rotation during anomerization.

Overall, these findings clarify the mechanistic role of Sn-based promoters in glycosylation and anomerization and provide a framework for the rational design of more efficient Lewis acid systems in carbohydrate chemistry.

## Supplementary Material



## Notes

Experimental procedures, computational details, notes about ^119^Sn NMR, summarized DFT results, additional tables and figures, NMR spectral data, as well as Cartesian coordinates (PDF)
