# Supplementary material for: Dissecting Tin-Based Activation and Anomerization Pathways in Carbohydrate Chemistry
Source: ACS Omega. 2026 Jan 20;11(4):5905–12. doi: 10.1021/acsomega.5c10211 (PMC12878332; doi:10.1021/acsomega.5c10211)
Supplement: Supplementary file 1 [file ao5c10211_si_001.pdf]

Dissecting Tin-Based Activation and Anomerization Pathways in Carbohydrate Chemistry

Claudio D. Navo,[a] María J. Moure,[a] Pablo Valverde,[b] Ana Poveda,[a] Gonzalo Jiménez-Osés,[a,c] Jesús Jiménez-Barbero,*[a,c,d,e] and Antonio Franconetti*[f]

[a] Center for Cooperative Research in Biosciences (CIC bioGUNE), Basque Research and Technology Alliance (BRTA), Derio,Bizkaia, 48160, Spain.

[b] Instituto de Investigaciones Químicas – CIC Cartuja, Avda. Americo Vespucio, 42, 41092, Sevilla, Spain.

[c] Ikerbasque, Basque Foundation for Science, Bilbao, 48009, Spain.

[d] Department of Organic & Inorganic Chemistry, Faculty of Science and Technology, University of the Basque Country, EHU-UPV, Leioa,Bizkaia, 48940, Spain.

[e] Centro de Investigacion Biomedica En Red de Enfermedades Respiratorias, Madrid, 28029, Spain.

[f] Departament de Química, Universitat Autònoma de Barcelona, 08193 Cerdanyola del Vallès, Spain.

*Corresponding authors: jjbarbero@cicbiogune.es and antonio.franconetti@uab.cat

Table of content:

1. Material and Methods
2. Synthesis of compounds **12β** and **12α**
3. Computational details
4. Notes about ^{119}Sn NMR. *Electronegativity of substituents*
5. Descriptors characterizing the formation of oxocarbenium ion pairs (Fig. S1)
6. Coordination preference of $SnCl_4$ (Fig. S1 and Table S1)
7. Energies, entropies, and lowest frequencies of the calculated structures (Table S1)
8. NMR spectral data
9. Cartesian coordinates
10. References

Material and Methods

General methods

NMR data were recorded on a Bruker NEO 400 spectrometer equipped with a BBO iProbe, at a frequency of 400.13 MHz for ^{1}H , 100.6 MHz for ^{13}C and 149.2 MHz for ^{119}Sn, and using TopSpin 4.1.1 (BRUKER) for both data acquisition and processing. Chemical shifts are reported in parts per million (ppm) relative to tetramethylsilane (TMS) as the internal standard for ^{1}H and ^{13}C signals, and ^{119}Sn signals are reported relative to tetramethyltin. NMR data is represented as follows: chemical shift, multiplicity (s = singlet, d = doublet, t = triplet, dd = doublet of doublets, m = multiplet and/or multiple resonances, bs. = broad signal), J coupling, integration and peak intensity. NMR signals were assigned on the basis of ^{1}H NMR, ^{13}C NMR, HSQC and ^{119}Sn NMR, experiments. Thin layer chromatography (TLC) was conducted on Silica gel 60 F254 (EMD Chemicals, Inc.) with detection by UV-absorption (254 nm) where applicable. Visualization of TLC plates were accomplished by spraying with 10% sulphuric acid in ethanol and Hanessian´s Stain, followed by charring at ~150 ºC. Reagents were purchased from commercial sources and used without further purification.

Synthesis

Methyl 2,3,4,6-tetra-*O*-acetyl-β-D-glucopyranoside (12β).[1]

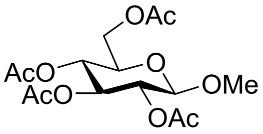

Methyl β-D-glucose (500 mg, 2.60 mmol) was dissolved in pyridine (3.55 mL) and the resulting solution was cooled to 0 ºC. 4-Dimethylaminopyridine (35 mg) and acetyl anhydride (2.2 mL) were added and the resulting suspension was then allowed to warm up to room temperature and stirring was continued for 18 h. After completion of the reaction, the reaction mixture was concentrated *in vacuo*. The residue was purified by silica gel column chromatography using hexane/ethyl acetate (8:2) as eluent to afford an amorphous solid (900 mg, 95%). ^{1}H NMR (400 MHz, $CDCl_3$): 5.00 (t, J = 9.4 Hz, 1H, H-3), 4.86 (t, J = 9.70 Hz, 1H, H-4), 4.74 (t, J = 8.72 Hz, 1H. H-2), 4.29 (d, J = 7.97 Hz, 1H, H-1), 4.09 (dd, J = 12.3, 4.65 Hz, 1H, H-6), 3.93 (d, J = 12.6 Hz, 1H, H-6'), 3.58-3.55 (m, 1H, H-5), 3.29 (s, 3H, OCH_3), 1.87, 1.83, 1.82 and 1.78 (s, 4 × $COCH_3$); ^{13}C NMR (400 MHz, CDCl3): 101.1 (C-1), 72.5 (C-3), 71.4 (C-5), 71.0 (C-2), 68.1 (C-4), 61.6 (2 × C-6), 56.5 (OCH_3) and 20.4 ($COCH_3$). MALDI-MS: *m*/*z* for $C_{15}H_{22}NaO_{10}$ [M+Na]+: 384.9105; found 384.801.

Methyl 2,3,4,6-tetra-*O*-acetyl-α-D-glucopyranoside (12α).

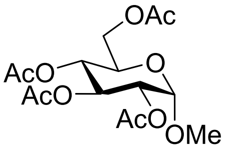

Methyl β-D-glucopyranoside tetraacetate (0.05 mmol, 20 mg) was dissolved in dichloromethane (600 μL) and stirred under argon for 10 min at r.t. After that, $SnCl_4$ (0.03 mmol, 7.2 mg) was added and the resulting mixture was stirred for 24 h at 45 ºC. After completion of the reaction, the reaction mixture was monitored by 1H NMR spectroscopy, using a 400 MHz NMR spectrometer at 45 ºC. ^{1}H NMR (400 MHz, $CDCl_3$): 5.46 (t, *J* = 10.0 Hz, 1H, H-3), 5.06 (t, *J* = 9.40 Hz, 1H, H-4), 4.90 (dd, *J* = 10.5, 3.73 Hz, 1H. H-2), 4.95 (d, *J* = 3.98 Hz, 1H, H-1), 4.27 (dd, *J* = 12.1, 4.43 Hz, 1H, H-6), 4.12 (d, *J* = 12.7 Hz, 1H, H-6'), 4.02-3.97 (m, 1H, H-5), 3.42 (s, 3H, OCH_3), 2.19, 2.12, 2.01, 2.05 and 2.02 (s, 4 × $COCH_3$). ^{13}C NMR (400 MHz, $CDCl_3$): 96.6 (C-1), 70.1 (C-3), 67.1 (C-5), 70.7 (C-2), 68.6 (C-4), 62.1 (2 x C6), 55.4 (OCH_3) and 20.6 ($COCH_3$).

Quantum mechanical calculations. Full geometry optimizations and transition structure (TS) searches were carried out with Gaussian 16[2] using the ωB97x-D functional[3] and def2-SVP[4] basis set and LANL2DZ[5] effective core potential for tin with ultrafine integration grids. Bulk solvent effects in dichloromethane were considered implicitly through the IEF-PCM polarizable continuum model.[6] The possibility of different conformations was taken into account for all structures. All stationary points were characterized by a frequency analysis performed at the same level used in the geometry optimizations from which thermal corrections were obtained at 298.15 K. The quasiharmonic approximation reported by Truhlar *et al.* was used to replace the harmonic oscillator approximation for the calculation of the vibrational contribution to enthalpy and entropy.[7] Scaled frequencies were not considered. Mass-weighted intrinsic reaction coordinate (IRC) calculations were carried out by using the Gonzalez and Schlegel scheme[8] in order to ensure that the TSs indeed connected the appropriate reactants and products. Gibbs free energies (ΔG) were used for the discussion on the relative stabilities of the considered structures. Free energies calculated using the gas phase standard state concentration (1 atm = 1/24.5 M) were converted to reproduce the standard state concentration in solution (1 M) by adding or subtracting 1.89 kcal mol^{-1} for bimolecular additions and decompositions, respectively. The lowest energy conformer for each calculated stationary point was considered in the discussion; all the computed structures can be obtained from authors upon request. Cartesian coordinates, electronic energies, entropies, enthalpies, Gibbs free energies, and lowest frequencies of the calculated structures are summarized in Table S1. The MEP (Molecular Electrostatic Potential) calculations have been performed at the ωB97x-D/def2-SVP (LANL2DZ for Sn) level of theory. The σ-hole value corresponds to the maximum positive electrostatic potential on

the Sn atom along the extension of the Sn–X bond. This procedure follows the standard approach implemented in GaussView for visualizing and extracting MEP data.

Calculation of theoretical ^{119}Sn chemical shifts. Model molecules displaying different coordination spheres ($SnCl_4$, $SnCl_5^-$, $SnCl_6^{2-}$, $SnCl_4(Et_2O)_2$ and $SnCl_4(H_2O)$ were calculated to compared with unknown coordination modes on methyl per-*O*-acetylated glucopyranoside. These small molecules were optimized (tight convergence criteria) using ωB97x-D functional and def2-QZVPPD[9] for all elements. Then, isotropic NMR shielding constants were calculated using Single Origin method (our results are similar than IGAIM)[10] as implemented in Guassian16 at B3LYP level.[11] The best results were provided by TZPall-s basis set for Sn atoms and 6-311++G(d,p) for all other elements. A superfinegrid was selected for these calculations. In addition, the Truhlar and coworkers' SMD solvation model[12] was applied using $CDCl_3$ as solvent. A good linear regression (r^2 = 0.9929) between experimental and theoretical values was obtained.

Calibration of calculated NMR shieldings to experimental chemical shifts via linear regression is a widely adopted approach for accurate prediction. Representative studies include ^{31}P,[13] ^{19}F,[14] ^{29}Si,[15] and ^{119}Sn.[16]

Notes about ^{119}Sn Nuclear Magnetic Resonance

Nuclear Magnetic Resonance (NMR) has become essential to study carbohydrate conformations and interactions.[17] In fact, this technique is a fundamental tool in the glycoscience field. This section is aiming to gather basic but relevant features of this nucleus to capture the attention for its further applications in glycosciences.

Sn(IV) species display a high structural variability due to the intrinsic ability of this element to adopt several coordination geometries in the presence of different donor ligands (usually from 4 to up to 7) under different experimental conditions (solid/solution, solvent or temperature, among others).[18] The coordination sphere is one of the most relevant factors affecting its NMR chemical shifts. As reported, higher coordination numbers induce great upfield shifts in δ(^{119}Sn). In some cases, the ^{119}Sn chemical shifts can decrease two/three hundreds of ppms from four- to five-coordinated Sn atoms and from five- to six-coordinated Sn atoms. To illustrate this point, the progressive treatment of $SnCl_4$ (in CH_2Cl_2, δ(^{119}Sn) = -160 ppm) with Ph_3CCl first shifts its signal to -460 ppm, which correspond to the pentacoordinated $SnCl_5^-$ species in fast exchange, and finally enables the visualization of another species in slow exchange at -723 ppm, which is the hexacoordinated $SnCl_6^{2-}$ anion.[19] For organotin compounds other than pure halides the upfield shifts are likewise remarkable and often range around 60-150 ppm between four- and five-coordinated Sn and 130-200 ppm between five- and six-coordinated Sn.[20]

It is important to stress that temperature and concentration usually do not affect much the ^{119}Sn chemical shifts of the same Sn(IV) complexes. Similarly, negligible changes in δ(^{119}Sn) are detected between non-polar solvents.[21] Worth noting, not all organotin compounds fulfil these conditions: in general, strong modifications on the δ(^{119}Sn) values when varying the temperature or the

concentration indirectly arise from changes in the coordination sphere. Likewise, polar solvents often give rise to important changes in δ(^{119}Sn) values of tetracoordinated tin (IV) compounds, as the donor solvent molecules can increase the Sn coordination number to 5 or 6. Some reported cases include pyridine,[22a] DMSO[22b] and H_2O/acetone mixtures.[22c] On the other hand, large chemical shift perturbations due to concentration changes are often ascribed to self-association phenomena, which also modify the metal coordination sphere.[23] Finally, temperature also plays a similar role: increasing the temperature drives a downshift effect in the ^{119}Sn resonances, as a result of dimer dissociation or release of bound solvent molecules, that decreases the coordination number.[24]

Aside from the coordination sphere, the proper nature of the donor ligands surrounding the Sn atom strongly affects the chemical shifts of the metal center, and may depend in certain cases on the electronegativity of the ligands (see the next section for an accurate description).[25] This rule of electronegativity usually works when the nature of a given substituent is modified without varying its number around the tin center. In general, an increase on the electron-withdrawing character of a substituent yields higher experimental ^{119}Sn chemical shifts.

Interestingly, chelating ligands can further modify ^{119}Sn resonances according to their geometry and the stability of the chelating ring. Since the ability to chelate becomes progressively worse as the ring is more constrained, a deshielding effect in the ^{119}Sn nucleus is observed from six-membered to analogous five- and/or four-membered chelating ligands.[20,26] Similarly, the arrangement of the donor ligands in a five-coordinated Sn complex, which has a trigonal bipyramid geometry, could be modified by the introduction of a bidentate chelating ligand, which may occupy one of the axial and one of the equatorial positions, displacing the bulkiest substituents from the equatorial plane. However, reported cases have proven that these differences in chemical shifts are not so remarkable in comparison to changes in the Sn coordination number, but instead they have an important effect in the $J(^{13}C-^{119}Sn)$ coupling constants.[27]

Electronegativity of substituents on ^{119}Sn NMR

Regarding the nature of a given substituent, the paramagnetic contribution is commonly taken as the most relevant effect triggering changes in the electronic environment.[28] Also, under certain assumptions, the paramagnetic contribution given by Eq. 1[29] should be mostly dependent on the electronegativity of the substituents.

$$\sigma^P = -\frac{1}{\Delta E}\left[A\langle r^{-3}\rangle_{5p}Q_{5p} + B\langle r^{-3}\rangle_{5d}Q_{5d}\right] \qquad \text{(Eq. 1)}$$

Thus, in the series $Me_{4-n}S_nX_n$ a downfield effect would be expected as the methyl groups are progressively substituted by the more electronegative X group. However, experimental evidences have revealed a more complex scenario: the first substitution produces the expected downfield effect, but then, the subsequent substitutions lower the δ(^{119}Sn) values. This has been observed for many *n*-methyltin derivatives $Me_{4-n}S_nX_n$, where X is chloride, bromide, iodide, amine, alkoxide, thioalkoxide

or selenoxide.[22b,30] The chemical shift variations depend on each substituent, for instance the ^{119}Sn resonates 100-150 ppm at higher field in $SnCl_4$ and $Sn(NEt_2)_4$ respect to Me_4Sn, whereas the difference is even higher in $Sn(OtBu_2)_4$, which shows a δ(^{119}Sn) of nearly -200 ppm compared to 0 ppm of Me_4Sn.[31]

Figure S1. Descriptors characterizing the formation of oxocarbenium ion pairs (**4**-**6**) from glycosyl halides (**1**-**3**): (a) Correlation between ρ(r) at the critical point for C—halide bond (*i.e.* 0.1600 a.u. for compound **1**) and C-O bond length. Two promoters were evaluated for each glycosyl donor; (b) Condensed dual descriptors (Δf_{C1}) for the anomeric carbon. A feasible nucleophilic attack should display $\Delta f_{C1} > 0$, whereas $\Delta f_{C1} < 0$ values present the opposite effect. Naked oxocarbenium species ($R_{C\text{-}O}$ = 1.25 Å, Δf_{C1} = 0.289) were employed as reference. Relevant distances (in Å) are also shown.

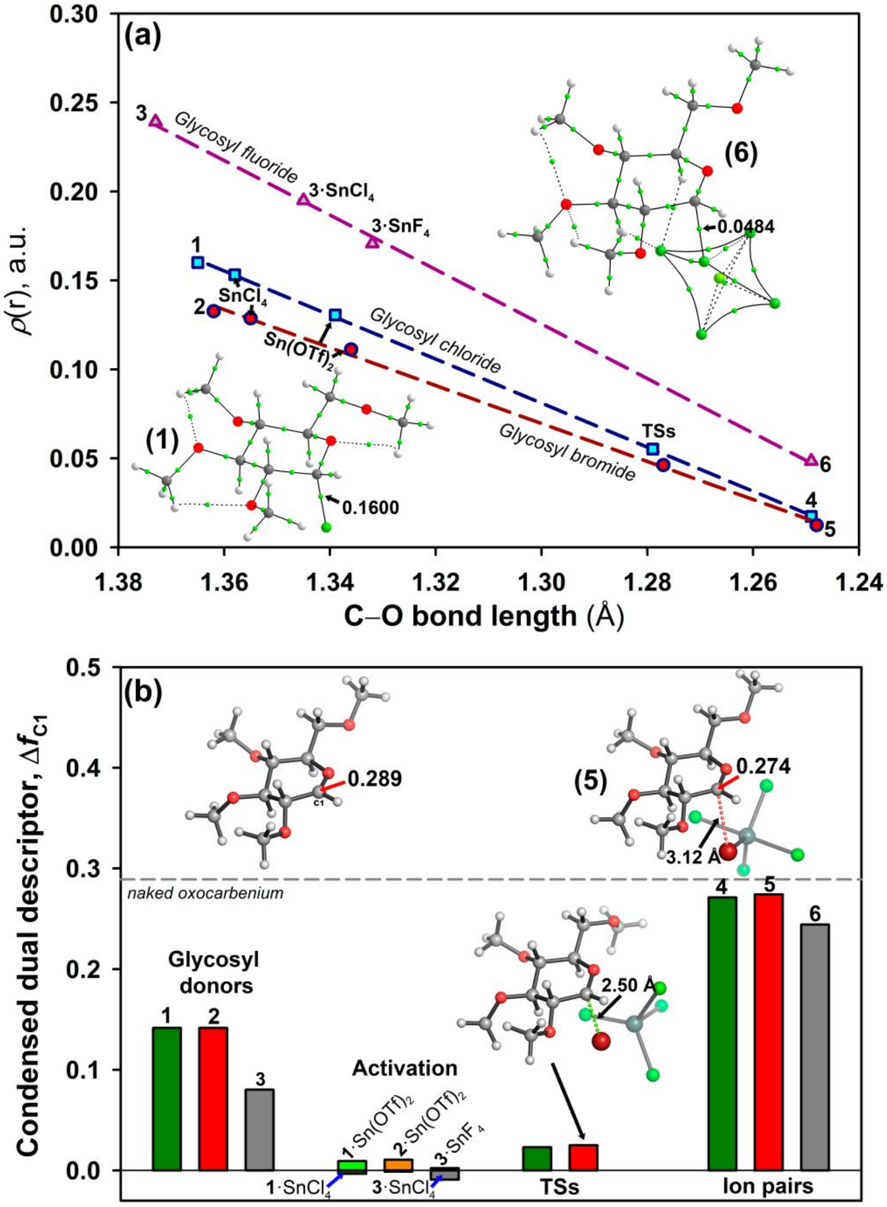

Figure S2. Free energy profile (ΔG, kcal·mol^{-1}) for glycosyl chloride activation and nucleophilic substitution with MeOH. The S_N2 transition state exhibits a high barrier, whereas oxocarbenium formation occurs with a significantly lower barrier, supporting the preferential S_N1 pathway under the studied conditions. The activation step for S_N1 is not shown. Energies are calculated at PCM(CH2Cl2)/ωB97X-D/def2-QZVPPD//ωB97X-D/def2-SVP (LanL2DZ for Sn atoms).

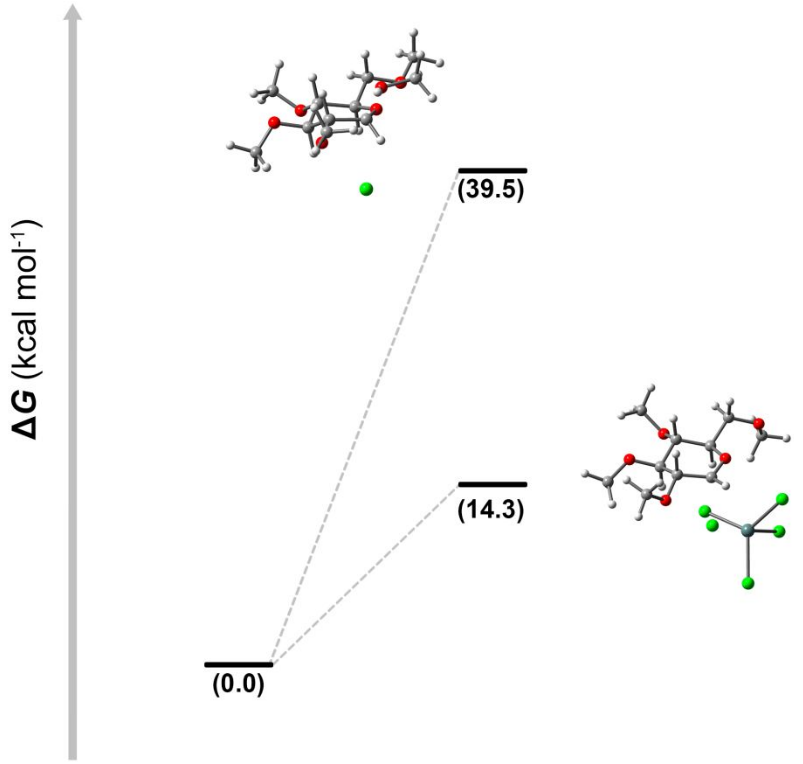

Figure S3. Calculated structures for the coordination to $SnCl_4$ and their corresponding Gibbs free energies (kcal mol^{-1}) at PCM(CH_2Cl_2)/ωB97x-D/def2-SVP level (LANL2DZ for Sn atom).

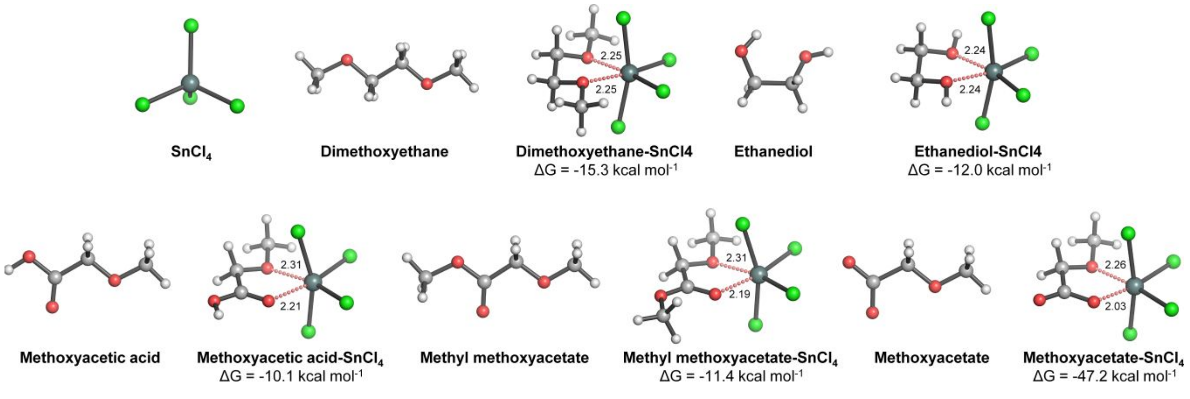

Figure S3. Optimized structures and activation barriers (ΔG‡) for the anomerization process and relative stability of the α anomer (ΔGα) with respect to the β anomer (arbitrarily set to 0) for different glycoside models calculated at PCM(CH_2Cl_2)/ωB97X-D/def2-SVP (LANL2DZ for Sn atoms)

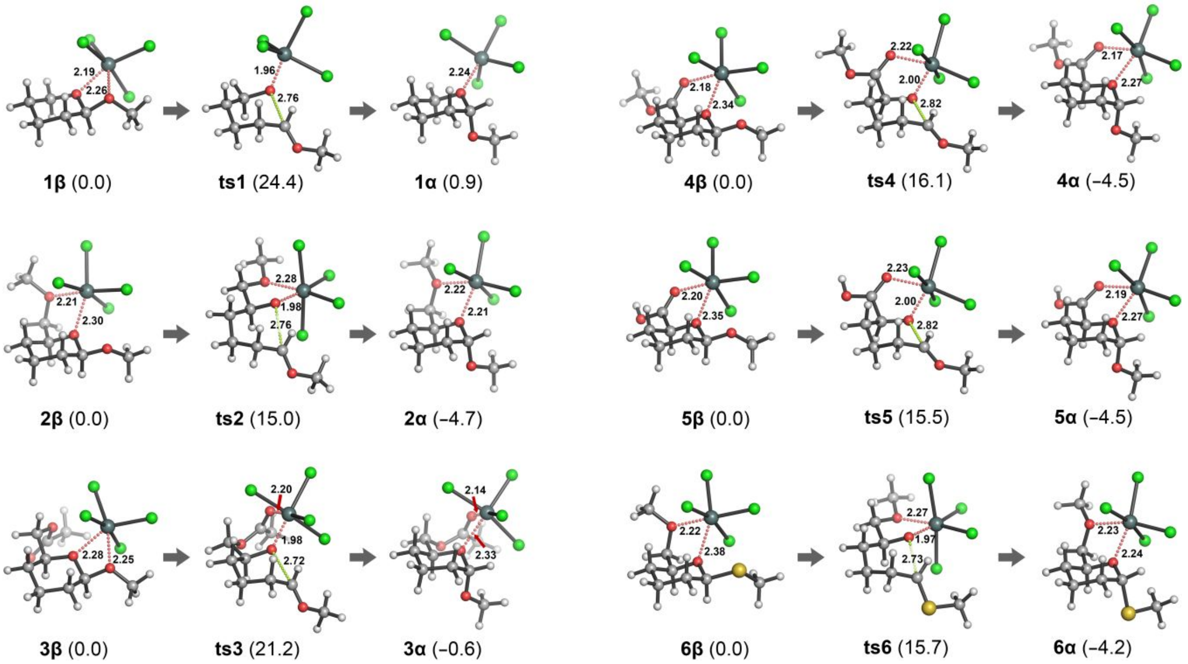

Table S1. Relative stability of Sn coordination, energies and ^{13}C and ^{119}Sn chemical shifts.

**Anomer**	**Structure**	**E_{elec}** (Hartree)	**E_{elec} + ZPE** (Hartree)	**G** (Hartree)	***N* of imag freq.**	**ΔE_{ZPE} (kcal/mol)**	**δ_{CO}** (ppm)	**$^{a}\delta_{CO}$** (ppm)	**$\Delta\delta_{CO}$** (ppm)	**Calcd. δ_{Sn}** (ppm)
β	C-2	-3179.559218	-3179.173146	-3179.248728	0	3.15	169.4	170.3	0.9	-493.9
	C-3	-3179.561223	-3179.174873	-3179.249344	0	2.07	170.3	170.8	0.5	-483.7
	C-4	-3179.559473	-3179.172956	-3179.247534	0	3.27	169.4	170.0	0.6	-475.4
	C-6, gg (I)	-3179.564801	-3179.178171	-3179.253171	0	0.00	170.7	172.3	1.6	-490.4
	C-6, gg (II)	-3179.548331	-3179.162330	-3179.239274	0	9.94				-486.3
	C-6, gt (I)	-3179.558375	-3179.172341	-3179.247815	0	3.66				-478.0
	C-6, gt (II)	-3179.560106	-3179.173736	-3179.248665	0	2.78				-505.9
	C-6, tg (I)	-3179.560179	-3179.173912	-3179.247685	0	2.67				-496.0
α	C-2	-3179.561652	-3179.175121	-3179.250637	0	3.20	-	170.9	-	-493.6
	C-3	-3179.563980	-3179.177504	-3179.252653	0	1.70	-	170.8	-	-483.8
	C-4	-3179.562309	-3179.175438	-3179.250916	0	3.00	-	170.4	-	-477.4
	C-6, gg (I)	-3179.566919	-3179.180217	-3179.255942	0	0.00	-	172.1	-	-490.9
	C-6, gg (II)	-3179.550972	-3179.164968	-3179.242811	0	9.57				-487.1
	C-6, gt (I)	-3179.561025	-3179.174840	-3179.251687	0	3.37				-479.8
	C-6, gt (II)	-3179.564654	-3179.177889	-3179.253192	0	1.46				-504.7
	C-6, tg (I)	-3179.562708	-3179.175707	-3179.249613	0	2.83				-496.8

[a]Coordinated with $SnCl_4$ (0.5 eq)

Table S2. Energies, entropies, and lowest frequencies of the lowest energy calculated structures.[a]

Structure	E_{elec} (Hartree)[b]	E_{elec} (Hartree)	E_{elec} + ZPE (Hartree)	H (Hartree)	S (cal mol^{-1} K^{-1})	G (Hartree)	Lowest freq. (cm^{-1})	# of imag freq.
$SnCl_4$	-	-1843.763366	-1843.758643	-1843.749973	90.4	-1843.792828	94.5	0
Dimethoxyethane	-	-308.536718	-308.394494	-308.385593	86.9	-308.426500	67.4	0
Dimethoxyethane-$SnCl_4$		-2152.354299	-2152.203143	-2152.186096	129.4	-2152.246725	60.9	0
Ethanediol	-	-230.017025	-229.930310	-229.924442	69.4	-229.957409	191.1	0
Ethanediol-$SnCl_4$	-	-2073.826555	-2073.731921	-2073.717813	116.5	-2073.772384	55.7	0
Methoxyacetic acid	-	-343.241645	-343.145592	-343.137932	81.5	-343.176246	71.1	0
Methoxyacetic acid-$SnCl_4$	-	-2187.049103	-2186.945182	-2186.928971	128.1	-2186.988144	32.5	0
Methyl methoxyacetate	-	-382.500780	-382.376876	-382.367492	90.7	-382.409757	56.2	0
Methyl methoxyacetate-$SnCl_4$	-	-2226.310878	-2226.178909	-2226.160967	136.4	-2226.223758	34.9	0
Methoxyacetate	-	-342.748099	-342.665238	-342.657898	80.0	-342.695686	80.1	0
Methoxyacetate-$SnCl_4$	-	-2186.615419	-2186.524127	-2186.508373	125.1	-2186.566671	45.7	0
I-β	-2441.838111	-2229.714113	-2229.526235	-2229.508421	134.6	-2229.570601	36.0	0
I-ts	-2441.794355	-2229.660770	-2229.476761	-2229.458101	142.4	-2229.522104	-151.2	1
I-α	-2441.836474	-2229.706336	-2229.518553	-2229.500738	135.2	-2229.563017	40.1	0
II-β-gg	-2595.699115	-2383.398975	-2383.148910	-2383.127657	147.9	-2383.196644	40.8	0
II-ts	-2595.670679	-2383.364239	-2383.117614	-2383.095635	154.1	-2383.166440	-103.1	1
II-α-gg	-2595.706704	-2383.407652	-2383.157434	-2383.136090	150.1	-2383.205204	32.0	0
II-β-gt	-2595.698320	-2383.394710	-2383.144577	-2383.123232	149.7	-2383.192445	37.0	0
II-α-gt	-2595.702258	-2383.401647	-2383.151373	-2383.130006	149.8	-2383.199209	34.8	0
III-β	-2709.076910	-2496.637917	-2496.378864	-2496.355257	164.1	-2496.429203	25.5	0
III-ts	-2709.040441	-2496.602352	-2496.345898	-2496.322262	161.7	-2496.396273	-197.5	1
III-α	-2709.080252	-2496.645826	-2496.385338	-2496.362586	155.1	-2496.434759	48.4	0
IV-β	-2669.753939	-2457.358070	-2457.126902	-2457.104837	153.7	-2457.175784	43.1	0
IV-ts	-2669.724358	-2457.324149	-2457.096212	-2457.073537	159.1	-2457.145844	-70.7	1
IV-α	-2669.761205	-2457.367368	-2457.136067	-2457.113907	156.3	-2457.185045	18.5	0

V-β	-2630.448961	-2418.096144	-2417.893073	-2417.872710	145.6	-2417.940070	45.2	0
V-ts	-2630.420383	-2418.063180	-2417.863327	-2417.842351	151.7	-2417.910974	-103.2	1
V-α	-2630.456264	-2418.105423	-2417.902225	-2417.881806	147.8	-2417.949250	20.2	0
VI-β	-2630.448961	-2706.320626	-2706.074745	-2706.052735	152.5	-2706.123426	35.5	0
VI-ts	-2630.420383	-2706.289733	-2706.047262	-2706.025078	154.3	-2706.096161	-101.0	1
VI-α	-2630.456264	-2706.333476	-2706.087285	-2706.065368	152.9	-2706.135681	34.9	0

[a]Energy values calculated at the PCM(CH_2Cl_2)/ωB97x-D/def2-SVP level and LANL2DZ for Sn. 1 Hartree = 627.51 kcal mol^{-1}. Thermal corrections at 298.15 K; [b] Single point calculations at the PCM(CH2Cl2)/ ωB97x-D/def2-QZVPPD

NMR spectral data

Compound 12β: ^{1}H NMR (400 MHz, $CDCl_3$)

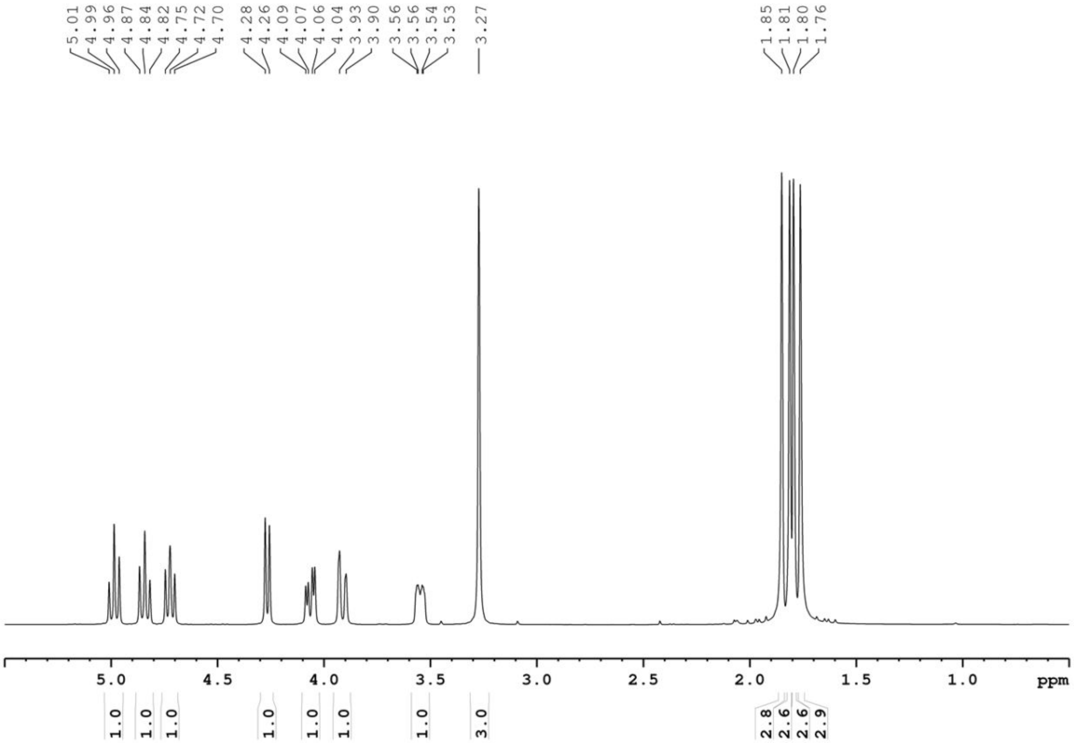

Compound 12β: HSQC (400 MHz, $CDCl_3$)

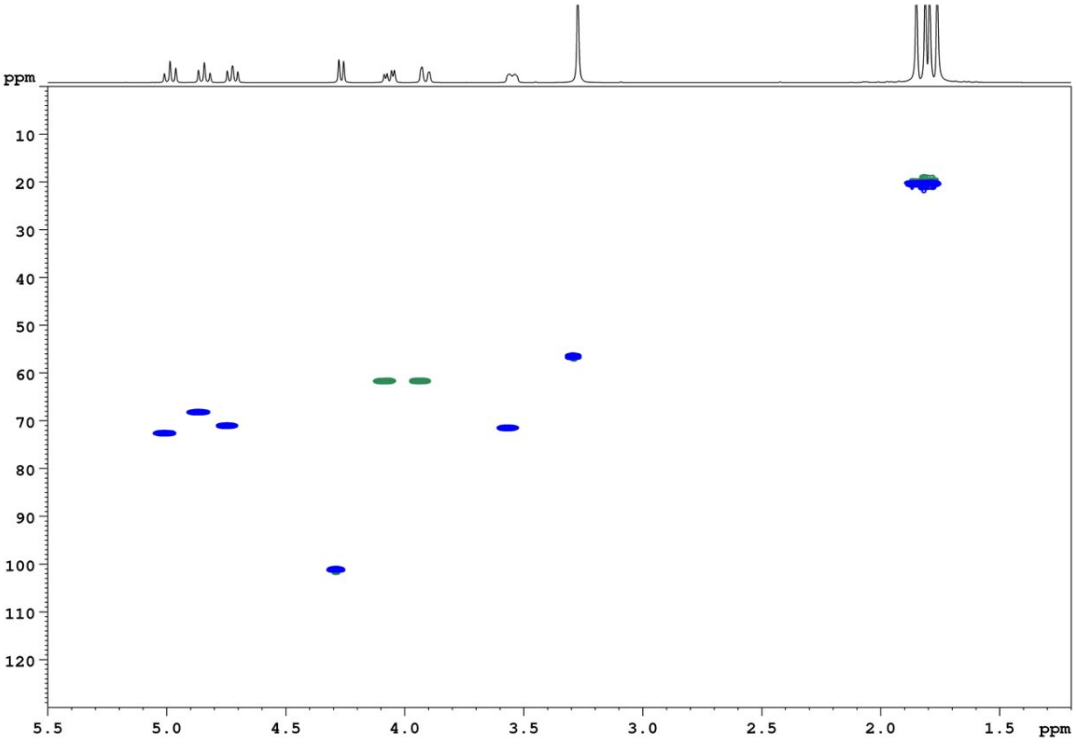

Compound 12α: HSQC (400 MHz, $CDCl_3$)

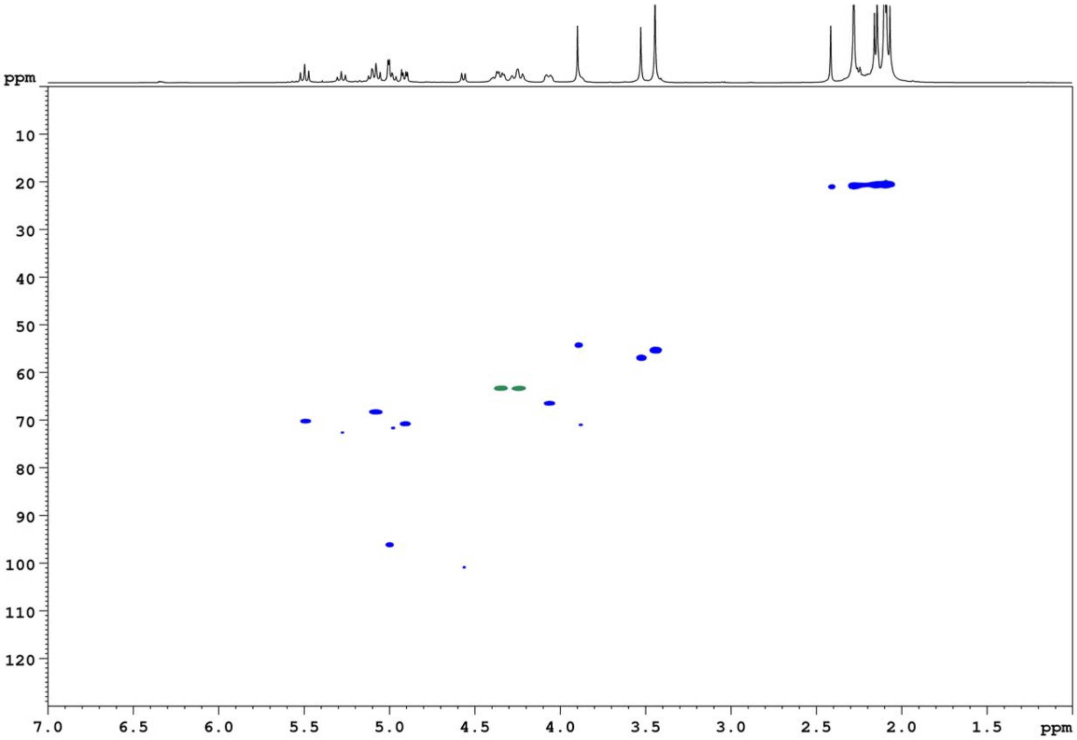

Complex $SnCl_4 \cdots OEt_2$: ^{119}Sn NMR (149 MHz, $CDCl_3$, 318 K).

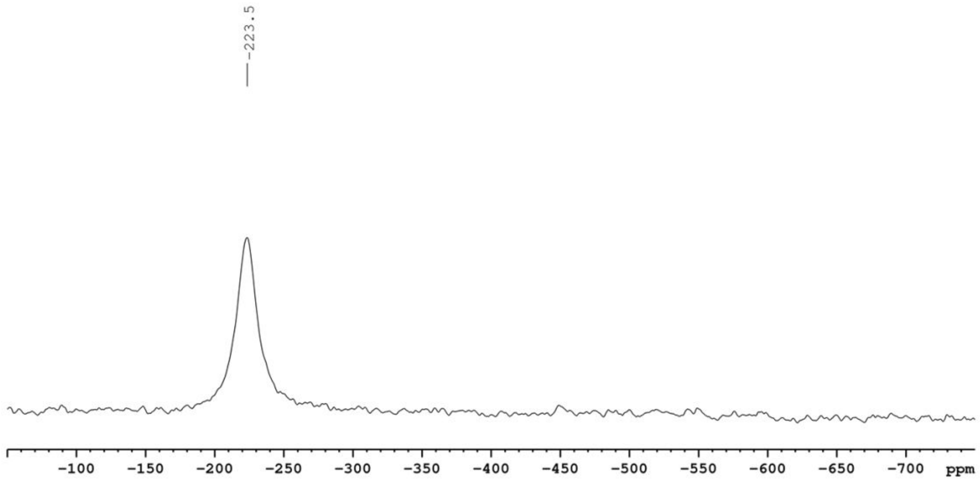

Complex $SnCl_4 \cdots OEt_2$: ^{119}Sn NMR (149 MHz, $CDCl_3$, 298 K).

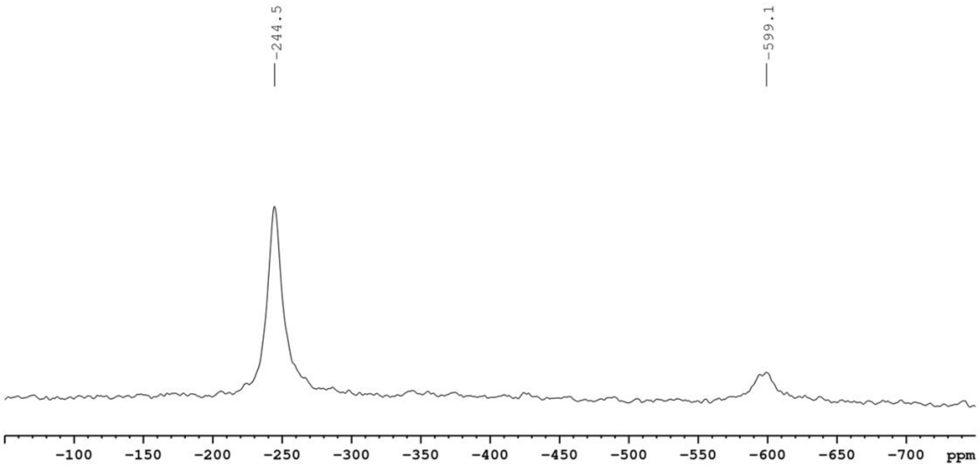

Complex $SnCl_4 \cdots OEt_2$: ^{119}Sn NMR (149 MHz, $CDCl_3$, 288 K).

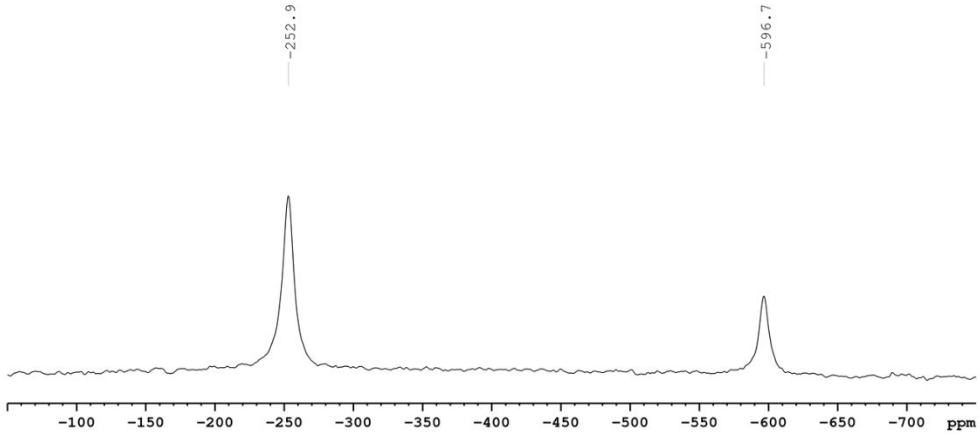

Complex $SnCl_4 \cdots EtOAc$ (*penta-coordinated*): ^{119}Sn NMR (400 MHz, $CDCl_3$, 298 K).

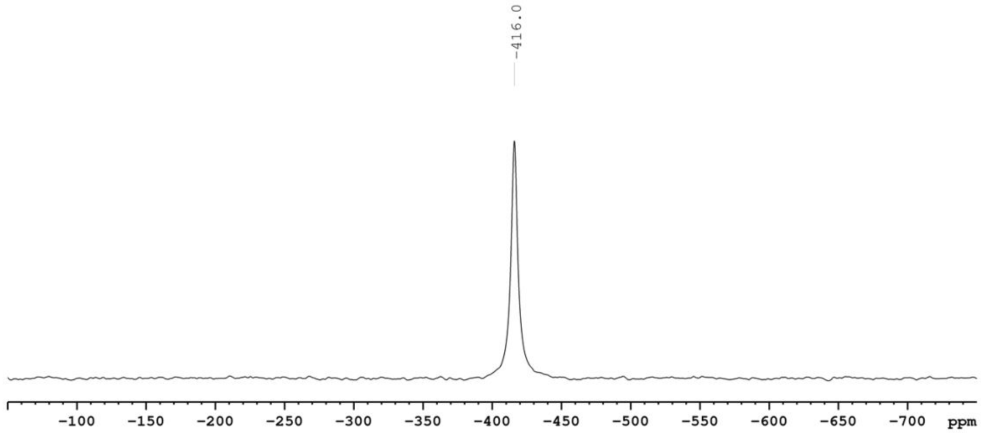

Complex $SnCl_4$··· $(EtOAc)_2$: ^{119}Sn NMR (149 MHz, $CDCl_3$, 318 K).

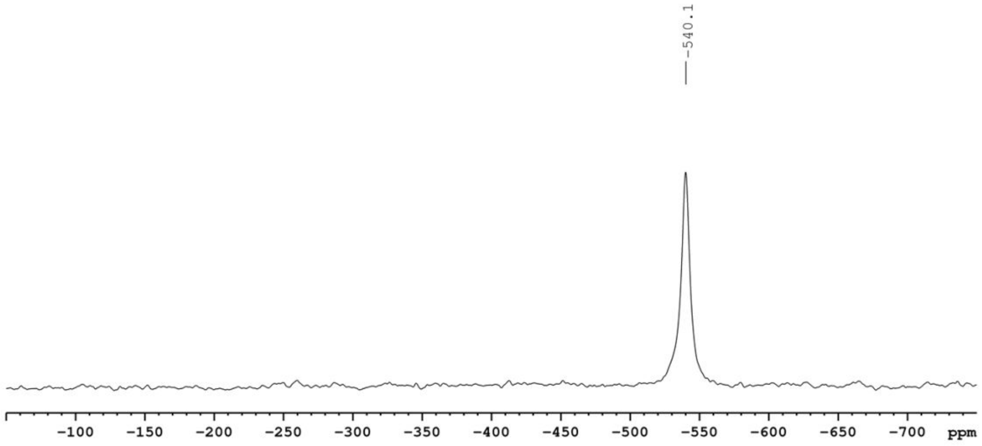

Complex $SnCl_4$··· $(EtOAc)_2$: ^{119}Sn NMR (149 MHz, $CDCl_3$, 298 K).

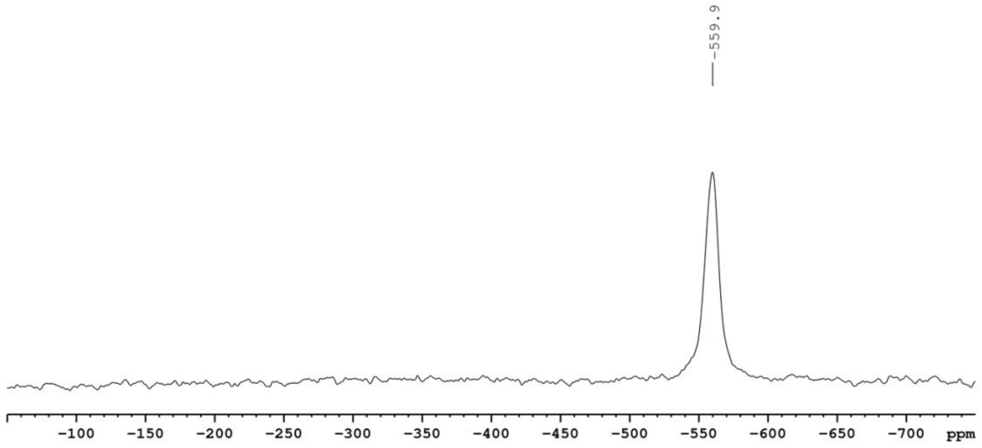

Complex $SnCl_4$··· $(EtOAc)_2$: ^{119}Sn NMR (149 MHz, $CDCl_3$, 288 K).

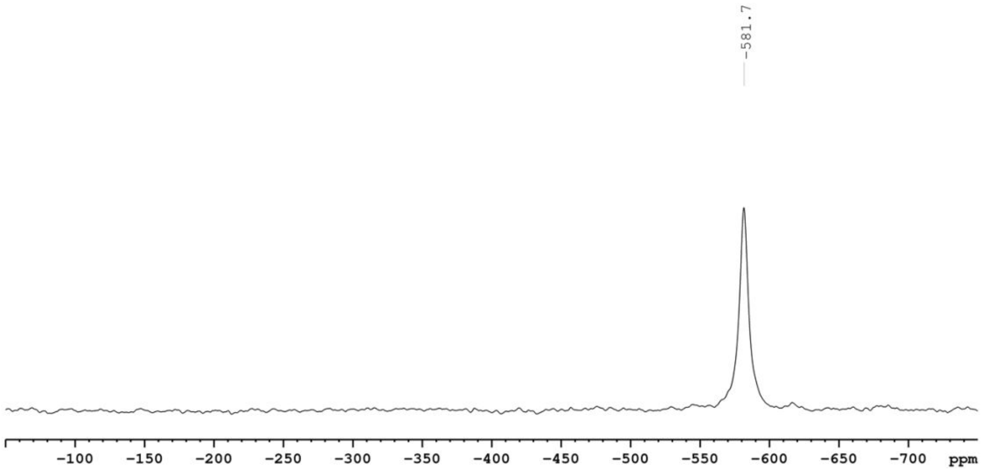

Cartesian coordinates of representative structures

Complex 4

C	-2.860130	0.476044	-0.479042
C	-1.831363	1.422388	0.147375
C	-1.531877	-0.177854	1.939419
C	-2.459755	-1.169298	1.336604
C	-2.522774	-0.985235	-0.186018
H	-0.917260	1.429552	-0.463990
H	-3.862368	0.690786	-0.058190
H	-1.053695	-0.375892	2.906727
H	-3.443373	-0.848273	1.755025
H	-1.526016	-1.206087	-0.604454
O	-1.341158	0.962984	1.468443
C	-2.354585	2.823535	0.338385
H	-2.801011	3.118678	-0.631620
H	-3.172009	2.814462	1.088996
O	-2.133355	-2.444827	1.749059
O	-3.497678	-1.820210	-0.725277
O	-2.831172	0.753665	-1.843349
O	-1.315864	3.661803	0.714166
C	-3.226706	-3.251139	2.147661
H	-2.822587	-4.245918	2.367538
H	-3.705821	-2.849356	3.056703
H	-3.974703	-3.329655	1.343968
C	-2.997284	-2.872756	-1.524229
H	-3.861533	-3.444026	-1.884775
H	-2.435942	-2.481850	-2.389086
H	-2.340664	-3.539703	-0.941453
C	-4.082135	0.738276	-2.496144
H	-4.754080	1.514210	-2.087874
H	-3.892089	0.956486	-3.554087
H	-4.570387	-0.243770	-2.407919
C	-1.704984	4.999162	0.877070
H	-2.469068	5.107746	1.669091
H	-0.813951	5.571267	1.163814
H	-2.113211	5.420408	-0.060067
Sn	2.230854	-0.175184	-0.183695
Cl	3.961997	-1.103598	-1.479240
Cl	0.804330	-1.609646	1.118306
Cl	3.646528	0.055753	1.741028
Cl	1.825205	2.152709	-0.218439
Cl	0.666085	-0.449683	-2.020151

Complex 5

C	2.993436	0.553251	0.455804
C	1.984562	1.456898	-0.258858
C	1.689031	-0.290400	-1.908254
C	2.551125	-1.261029	-1.185050
C	2.616319	-0.921168	0.309421
H	1.053603	1.513483	0.324258
H	3.997707	0.703743	0.012022
H	1.225107	-0.563882	-2.864986
H	3.555056	-1.044585	-1.622399
H	1.613479	-1.067634	0.745901
O	1.527940	0.895073	-1.554112
C	2.523315	2.835911	-0.542363
H	2.949235	3.198158	0.413883
H	3.358243	2.766384	-1.269813
O	2.153295	-2.552009	-1.466883
O	3.564623	-1.729072	0.931630
O	2.978785	0.957638	1.788376
O	1.501638	3.652257	-1.002977
C	3.199041	-3.461845	-1.757606
H	2.735637	-4.447953	-1.875781
H	3.710719	-3.190339	-2.696567
H	3.931418	-3.496888	-0.936994
C	3.043155	-2.610252	1.905336
H	3.889349	-3.172927	2.318367
H	2.546452	-2.055257	2.718234
H	2.323046	-3.316592	1.459815
C	4.235296	0.986943	2.430342
H	4.909537	1.720929	1.954123
H	4.055952	1.293042	3.468134
H	4.715573	-0.003135	2.420006
C	1.906247	4.972421	-1.247659
H	2.690743	5.020907	-2.025664
H	1.027567	5.529649	-1.595316
H	2.294963	5.454306	-0.331666
Sn	-2.218086	0.039184	0.285807
Cl	-3.882565	-0.525024	1.857934
Cl	-3.812756	0.056532	-1.517053
Cl	-1.691596	2.312306	-0.098727
Cl	-0.536204	-0.022362	2.052066
Br	-0.892728	-1.746308	-0.933789

Complex 6

C	2.266922	0.512270	0.265853
C	1.149008	1.412552	-0.260676
C	0.656599	-0.261306	-1.914566
C	1.601193	-1.263859	-1.341162
C	1.890231	-0.963523	0.128329
H	0.280568	1.341546	0.408517
H	3.187914	0.695937	-0.320752
H	0.152443	-0.480067	-2.860964
H	2.530308	-1.054677	-1.921702
H	0.965540	-1.137068	0.700661
O	0.635786	0.947015	-1.562164
C	1.561374	2.849669	-0.439463
H	2.063348	3.154061	0.499061
H	2.307147	2.924232	-1.257273
O	1.141498	-2.543934	-1.586586
O	2.935931	-1.776562	0.571654
O	2.458970	0.857600	1.604427
O	0.433213	3.619283	-0.698470
C	2.129245	-3.470882	-1.990822
H	1.630092	-4.441365	-2.096850
H	2.567192	-3.187344	-2.963874
H	2.931310	-3.551421	-1.241805
C	2.649982	-2.521506	1.734947
H	3.555105	-3.087611	1.988857
H	2.388586	-1.862375	2.579097
H	1.821466	-3.229565	1.562736
C	3.803861	0.971444	2.011875
H	4.315445	1.794030	1.480757
H	3.797132	1.195632	3.085675
H	4.357188	0.034600	1.840808
C	0.709370	4.984850	-0.852015
H	1.386985	5.171004	-1.706137
H	-0.242311	5.498369	-1.037461
H	1.172779	5.410954	0.057172
Sn	-2.270132	-0.286991	0.337532

F	-2.427170	1.558868	0.448727
F	-0.799873	-0.240741	1.532948
F	-1.003541	-0.959832	-0.942700
F	-3.552737	-0.437126	-1.012130
F	-3.217957	-1.383924	1.488953

Complex 9·$SnCl_4(CH_3CO_2)$

C	3.082098	-1.028016	-0.129952
C	3.351888	0.463942	0.066059
C	1.054478	0.938384	0.365003
C	0.925572	-0.406236	1.116783
C	1.975796	-1.485551	0.819375
H	3.576780	0.694024	1.122992
H	2.738941	-1.157587	-1.166558
H	-0.086759	-0.800350	0.973485
H	2.439768	-1.751416	1.781489
O	2.170740	1.139342	-0.356716
O	1.011808	-0.013305	2.512193
C	0.986243	1.261217	2.607204
O	1.010199	1.899965	1.507745
C	0.911746	1.933607	3.907679
H	-0.158600	2.101417	4.111286
H	1.325114	1.289962	4.690870
H	1.415454	2.904835	3.858331
C	4.494029	0.971095	-0.781383
H	5.383633	0.357227	-0.578172
H	4.221889	0.873851	-1.844548
O	4.711365	2.318918	-0.421072
O	4.258416	-1.754167	0.134978
O	1.380048	-2.626237	0.238230
C	4.679169	-2.822821	-0.597740
O	5.669957	-3.383210	-0.225027
C	5.606090	3.099256	-1.062129
O	5.752858	4.225956	-0.673399
C	0.600993	-3.391571	1.041516
O	0.441430	-3.123781	2.202182
C	-0.017031	-4.521540	0.283518
H	-0.771378	-4.101531	-0.398488
H	0.737236	-5.040987	-0.321749
H	-0.497742	-5.214589	0.981414
C	3.889671	-3.207017	-1.818088
H	2.839839	-3.404145	-1.564026
H	3.915879	-2.402843	-2.568033
H	4.345169	-4.106084	-2.245003
C	6.350891	2.497927	-2.223628
H	6.893281	1.590559	-1.922081
H	5.655051	2.220677	-3.029031
H	7.062249	3.238908	-2.601874
H	0.180832	1.184529	-0.249209
Sn	-3.139925	0.559275	-0.316203
O	-4.513483	-1.071272	0.015547
C	-4.899281	-1.038076	-1.191860
O	-4.370930	-0.156936	-1.935077
C	-5.889620	-2.014021	-1.722003
H	-6.450365	-1.574456	-2.554220
H	-5.331316	-2.884678	-2.098102
H	-6.561000	-2.348660	-0.923660
Cl	-2.195634	0.609301	1.898883
Cl	-4.881135	2.085739	0.278168
Cl	-1.558295	-1.178325	-1.015163
Cl	-1.875133	2.202223	-1.498578

Complex 10·$SnCl_4(CH_3CO_2)$

C	-2.738117	1.081485	0.063545
C	-1.318795	1.654601	0.100480
C	-0.596052	-0.350850	1.241277
C	-1.929471	-0.997948	1.146904
C	-2.735169	-0.439841	-0.018374
H	-0.863659	1.621345	-0.900447
H	-3.304573	1.398589	0.953037
H	0.235217	-0.806917	1.791722
H	-2.430518	-0.722922	2.102415
H	-2.296639	-0.774610	-0.970459
O	-0.406331	0.838413	0.904535
C	-1.249071	3.057539	0.672806
H	-2.014968	3.669453	0.180825
H	-1.449744	3.035035	1.753563
O	-1.810903	-2.385772	1.083540
O	-4.055703	-0.905313	0.096596
O	-3.373615	1.568335	-1.096312
O	-0.002186	3.658121	0.400832
C	0.966270	3.591411	1.335844
O	0.799776	3.081551	2.414757
C	-2.493058	-3.115754	2.014830
O	-3.107274	-2.597468	2.904260
C	-4.625113	-1.512144	-0.980296
C	-4.359992	2.492758	-0.958770
O	-4.632738	2.989442	0.099789
C	2.239886	4.202940	0.840560
H	2.035396	5.120567	0.274784
H	2.909040	4.408199	1.683087
H	2.715405	3.487559	0.151782
C	-2.352052	-4.576484	1.740011
H	-1.290172	-4.834452	1.631503
H	-2.809439	-5.151935	2.550854
H	-2.847711	-4.810276	0.786835
C	-5.006430	2.773322	-2.277279
H	-5.780320	3.537648	-2.155310
H	-4.246848	3.110869	-2.995766
H	-5.444698	1.846209	-2.672548
C	-6.032564	-1.912963	-0.667833
H	-6.468414	-2.433750	-1.526311
H	-6.045075	-2.561133	0.219201
H	-6.623031	-1.016970	-0.430001
O	-4.042729	-1.679404	-2.014552
Sn	2.694954	-0.674916	-0.675417
O	2.262522	0.055643	1.325431
C	3.395220	0.622391	1.402350
O	4.152909	0.506238	0.393514
C	3.796858	1.371983	2.622062
H	4.701419	1.959984	2.436863
H	2.960878	2.011848	2.936201
H	3.988725	0.644807	3.424629
Cl	0.471927	-1.657544	-0.893341
Cl	3.515058	-2.637598	0.425683
Cl	1.804961	1.424152	-1.492326
Cl	3.830297	-1.110556	-2.700219

$SnCl_4$

Sn	0.000000	0.000000	0.000000
Cl	-1.331013	1.331013	1.331012
Cl	1.331013	-1.331013	1.331012

Cl	1.331013	1.331013	-1.331012
Cl	-1.331013	-1.331013	-1.331012

Dimethoxyethane

C	-0.646572	0.394657	-0.000000
H	-0.676535	1.052812	0.891680
H	-0.676535	1.052812	-0.891681
C	0.646572	-0.394657	0.000000
H	0.676535	-1.052812	-0.891680
H	0.676535	-1.052812	0.891681
O	-1.712630	-0.509175	-0.000000
O	1.712630	0.509175	-0.000000
C	2.966750	-0.108304	0.000000
H	3.112293	-0.743141	0.895124
H	3.112293	-0.743142	-0.895123
H	3.732844	0.678559	-0.000000
C	-2.966750	0.108304	0.000001
H	-3.732844	-0.678559	-0.000000
H	-3.112293	0.743141	0.895125
H	-3.112294	0.743142	-0.895122

Dimethoxyethane-$SnCl_4$

O	-1.383558	-1.282614	0.394450
C	-2.590620	-0.737760	-0.143665
C	-2.590625	0.737758	0.143638
H	-2.628286	-0.938128	-1.226115
H	-3.449699	-1.212581	0.350983
H	-3.449700	1.212574	-0.351021
H	-2.628304	0.938126	1.226088
O	-1.383558	1.282617	-0.394462
Sn	0.422477	-0.000000	-0.000001
Cl	0.072768	-0.737452	-2.246385
Cl	1.921454	-1.737291	0.593894
Cl	1.921448	1.737299	-0.593885
Cl	0.072751	0.737446	2.246382
C	-1.315190	-2.712523	0.313764
H	-0.405178	-3.023354	0.834711
H	-2.198913	-3.131875	0.812219
H	-1.278106	-3.026029	-0.739713
C	-1.315181	2.712524	-0.313736
H	-0.405181	3.023366	-0.834695
H	-2.198915	3.131895	-0.812156
H	-1.278073	3.025999	0.739750

Ethanediol

C	0.681636	0.607153	-0.253457
H	0.676243	0.633383	-1.358359
H	1.190754	1.519918	0.108139
C	-0.746931	0.573051	0.250735
H	-1.300556	1.442802	-0.134323
H	-0.738493	0.647115	1.356576
O	1.302168	-0.568149	0.223446
H	2.107328	-0.724194	-0.277698
O	-1.399180	-0.593414	-0.180053
H	-0.767404	-1.307743	-0.025153

Ethanediol-$SnCl_4$

O	-1.501096	-0.338194	1.264611
C	-2.732753	-0.516402	0.548908
C	-2.732695	0.516564	-0.549213
H	-2.771973	-1.534301	0.135131
H	-3.578721	-0.349719	1.227608
H	-3.578564	0.349846	-1.228029
H	-2.772005	1.534465	-0.135451
O	-1.500937	0.338418	-1.264771
Sn	0.314761	-0.000009	-0.000012
Cl	-0.027697	-2.290508	-0.591741
Cl	1.691103	-0.485115	1.857275
Cl	1.691776	0.484882	-1.856833
Cl	-0.027510	2.290563	0.591559
H	-1.398999	1.000534	-1.964930
H	-1.399279	-1.000123	1.964964

Methoxyacetic-acid

C	0.270889	-0.552192	-0.000008
H	0.329662	-1.215625	-0.886959
H	0.329655	-1.215608	0.886956
O	-2.051710	-0.833692	-0.000018
O	1.256086	0.421095	-0.000013
C	2.552811	-0.108405	0.000039
H	2.741884	-0.729752	0.895775
H	2.741933	-0.729814	-0.895643
H	3.257093	0.733331	0.000029
C	-1.094344	0.096913	-0.000020
O	-1.312703	1.277887	0.000004
H	-2.909745	-0.382743	-0.000011

Methoxyacetic acid-$SnCl_4$

O	1.249018	1.287651	0.362280
C	2.522366	0.764797	0.080484
H	2.884799	1.086739	-0.912058
H	3.254973	1.070786	0.843593
O	3.541309	-1.356457	0.034072
Sn	-0.538971	-0.127418	-0.007696
Cl	0.144725	0.275683	-2.270307
Cl	-2.117896	1.639049	0.004335
Cl	-1.793097	-2.070078	-0.438139
Cl	-0.433093	-0.321146	2.358167
C	1.151594	2.708850	0.210021
H	0.169998	3.000288	0.594537
H	1.944964	3.188415	0.798042
H	1.238749	2.974665	-0.853670
C	2.411822	-0.731749	0.064755
O	1.325253	-1.303870	0.062496
H	3.404873	-2.319627	-0.006947

Methyl methoxyacetate

C	0.758485	-0.536058	-0.000112
H	0.789143	-1.200970	-0.887164
H	0.788960	-1.200921	0.886986
O	-1.566279	-0.719355	-0.000051
O	1.786724	0.392798	-0.000028
C	3.057791	-0.195040	0.000192
H	3.218125	-0.824722	0.895824
H	3.218378	-0.824849	-0.895305
H	3.800506	0.613057	0.000238
C	-2.893644	-0.208427	0.000181
H	-3.557647	-1.078976	0.000397
H	-3.074625	0.404058	0.894588
H	-3.074994	0.403892	-0.894266
C	-0.577619	0.174147	-0.000288
O	-0.742686	1.364268	-0.000063

Methyl_methoxyacetate_SnCl₄

O	0.481440	1.727828	0.365530
C	1.862253	1.708613	0.099501
H	2.090763	2.152019	-0.885479
H	2.421484	2.253704	0.875419
O	3.578978	0.105432	0.050924
Sn	-0.648939	-0.248297	-0.006988
Cl	-0.173015	0.382120	-2.272522
Cl	-2.770153	0.811551	0.011265
Cl	-1.110406	-2.513606	-0.446712
Cl	-0.485822	-0.400173	2.360581
C	-0.130177	3.011597	0.194170
H	-1.154242	2.925872	0.568741
H	0.425441	3.755414	0.780044
H	-0.135743	3.280122	-0.872467
C	4.095872	-1.238319	-0.021278
H	5.182796	-1.130461	-0.044343
H	3.775953	-1.801465	0.863420
H	3.729765	-1.723638	-0.933866
C	2.306063	0.273511	0.075360
O	1.499131	-0.656673	0.060674

Methoxyacetate

C	0.210846	-0.547092	0.000025
H	0.309793	-1.208991	-0.886403
H	0.309824	-1.208896	0.886521
O	-1.363768	1.269248	-0.000083
O	1.212251	0.433488	-0.000038
C	2.497326	-0.098904	0.000035
H	2.691036	-0.727407	0.893641
H	2.691086	-0.727546	-0.893461
H	3.217662	0.732445	-0.000009
C	-1.227433	0.033594	0.000038
O	-2.111463	-0.850885	0.000011

Methoxyacetate-SnCl₄

O	1.344525	1.159517	0.477138
C	2.573029	0.533261	0.143185
H	2.940990	0.912383	-0.825276
H	3.321716	0.743280	0.921031
O	3.365238	-1.687955	-0.037214
Sn	-0.437896	-0.140451	-0.014733
Cl	0.148514	0.510476	-2.280130
Cl	-1.923085	1.746890	0.148999
Cl	-1.981612	-1.835317	-0.633920
Cl	-0.555808	-0.473705	2.366052
C	1.358309	2.580241	0.369237
H	0.407965	2.942145	0.773036
H	2.196507	2.978753	0.957294
H	1.454724	2.873985	-0.687286
C	2.387396	-0.976421	0.020801
O	1.160779	-1.393105	-0.042234

I-β

C	-1.721507	1.049407	0.342565
C	-2.214503	-1.284823	0.966161
C	-3.413839	-1.287680	0.040376
C	-3.991677	0.121367	-0.094488
C	-2.914804	1.094483	-0.581699
H	-3.101286	-1.663146	-0.946639
H	-4.162064	-1.986757	0.440991
H	-4.834205	0.125748	-0.799286
H	-4.389208	0.457766	0.878090
H	-2.558454	0.807046	-1.582387
H	-3.294177	2.125238	-0.629497
H	-2.489226	-1.047225	2.006097
O	-1.272026	-0.285052	0.529156
H	-1.911104	1.509971	1.328749
Sn	0.838945	-0.175937	-0.047403
Cl	0.009148	-0.717321	-2.222753
Cl	1.343462	-2.407259	0.523248
Cl	2.736025	0.967267	-0.860578
Cl	1.117268	0.643910	2.190195
O	-0.548804	1.600155	-0.227813
C	-0.245781	2.972210	0.044845
H	0.687285	3.186049	-0.486809
H	-1.051563	3.605928	-0.345984
H	-0.110894	3.120190	1.125225
H	-1.653394	-2.226694	0.952409

I-ts

C	-2.901565	0.559959	-0.374507
C	-1.025965	-1.726423	1.260168
C	-1.838721	-2.504197	0.229947
C	-3.195283	-1.917752	-0.156754
C	-3.188992	-0.675459	-1.100014
H	-1.229314	-2.656660	-0.674700
H	-2.026508	-3.503486	0.655574
H	-3.758679	-2.679791	-0.713293
H	-3.792746	-1.693239	0.742237
H	-2.397066	-0.807808	-1.853670
H	-4.169468	-0.581161	-1.585470
H	-1.631889	-1.598263	2.177149
O	-0.631025	-0.451440	0.826651
H	-1.872528	0.815053	-0.083263
Sn	1.090697	0.067680	0.042599
Cl	0.564910	-0.966346	-2.044820
Cl	3.134553	0.934637	-0.888727
Cl	0.300970	2.304230	0.357443
Cl	2.314977	-1.152210	1.673655
O	-3.844222	1.300484	-0.008465
C	-3.587219	2.508538	0.753060
H	-2.505915	2.640848	0.881354
H	-4.032919	3.329705	0.183594
H	-4.098685	2.381472	1.712221
H	-0.152681	-2.336292	1.553038

I-α

C	2.158296	0.641959	0.677171
C	1.745055	-0.846281	-1.214695
C	2.723396	-1.869473	-0.676247
C	3.794091	-1.205631	0.185246
C	3.129228	-0.360311	1.267678
H	2.166006	-2.612266	-0.083847
H	3.168612	-2.398417	-1.532200
H	4.444408	-1.963546	0.644639
H	4.434718	-0.563854	-0.440099
H	2.560637	-1.001127	1.959225
H	3.866690	0.197794	1.861796
H	2.226719	-0.135367	-1.900899
O	1.159552	-0.071846	-0.130046
H	1.558412	1.135200	1.453985
Sn	-1.072438	-0.050744	0.034171
Cl	-0.760062	-2.320899	0.497688

Cl	-3.438708	-0.158473	0.087813
Cl	-0.871407	1.493284	1.773213
Cl	-0.975399	0.772595	-2.155483
O	2.819899	1.545049	-0.108338
C	2.055176	2.664704	-0.508645
H	1.699514	3.232698	0.367301
H	2.707222	3.303877	-1.114801
H	1.186788	2.358029	-1.113396
H	0.909874	-1.321870	-1.741162

II-β-gg

C	-2.442781	0.894283	0.150499
C	-1.760026	-1.231248	1.075642
C	-2.474062	-2.000808	-0.027089
C	-3.620089	-1.173988	-0.609378
C	-3.152379	0.226678	-1.004315
H	-1.748336	-2.242191	-0.817686
H	-2.849636	-2.946759	0.390896
H	-4.051913	-1.686560	-1.480146
H	-4.426028	-1.090884	0.139659
H	-2.452425	0.189482	-1.851803
H	-4.002894	0.861673	-1.290253
H	-2.460260	-1.017573	1.901664
O	-1.303311	0.041308	0.565864
C	-0.551487	-1.922099	1.672159
H	-0.778320	-2.973658	1.887504
O	0.525554	-1.845833	0.735894
H	-3.083606	0.926383	1.054058
C	0.985201	-3.078089	0.165698
H	1.357959	-3.722200	0.972827
H	0.169007	-3.569082	-0.380975
H	1.803787	-2.830739	-0.516940
Sn	0.887130	0.168372	-0.108218
Cl	0.132766	-0.779450	-2.172739
Cl	3.156366	-0.503160	-0.391075
Cl	1.045309	2.357683	-0.995559
Cl	1.173091	0.850851	2.168771
H	-0.223002	-1.424311	2.592746
O	-1.979096	2.117110	-0.182036
C	-1.701851	2.985363	0.904400
H	-1.314756	3.917028	0.478197
H	-2.622279	3.194609	1.474160
H	-0.942182	2.554240	1.573773

II-ts

C	-3.182108	-0.419195	0.385517
C	-0.928674	1.817008	-0.358366
C	-2.074121	2.668235	0.170496
C	-3.465404	2.060360	-0.008999
C	-3.826653	0.813053	0.847538
H	-1.885436	2.891713	1.233152
H	-2.073288	3.630277	-0.367257
H	-4.213033	2.815497	0.271261
H	-3.645914	1.833160	-1.071844
H	-3.481117	0.981532	1.880423
H	-4.915238	0.669225	0.838983
H	-1.203959	1.442615	-1.366260
O	-0.678746	0.746210	0.506626
C	0.364992	2.602905	-0.517043
H	0.290049	3.366259	-1.306766
H	0.658006	3.077659	0.435787
O	1.358629	1.647576	-0.891658
H	-2.102670	-0.566541	0.555189
C	2.664779	2.186422	-1.066806
H	2.633487	2.964927	-1.842203
H	3.032950	2.604617	-0.117934
H	3.311514	1.363488	-1.387902
Sn	0.923575	-0.344740	0.123413
Cl	2.075452	0.636046	2.014975
Cl	2.977024	-1.247398	-0.731950
Cl	0.063871	-2.231499	1.343063
Cl	-0.168509	-1.031311	-1.966140
O	-3.847530	-1.274141	-0.240627
C	-3.227470	-2.497286	-0.722321
H	-3.858298	-3.313974	-0.359058
H	-3.250020	-2.442629	-1.815080
H	-2.199910	-2.566279	-0.345080

II-α-gg

C	2.292580	-0.810177	-0.385019
C	1.697370	1.192007	0.891170
C	2.335839	2.100101	-0.146839
C	3.474502	1.367818	-0.855480
C	2.984612	0.040344	-1.429234
H	1.563255	2.410503	-0.867152
H	2.708136	3.001800	0.361869
H	3.886096	1.992591	-1.660509
H	4.292639	1.182247	-0.141305
H	2.256519	0.219556	-2.233685
H	3.811748	-0.549823	-1.848174
H	2.447087	0.880693	1.633988
O	1.218457	-0.017777	0.248511
C	0.522327	1.787546	1.636216
H	0.743669	2.832741	1.892336
O	-0.607189	1.753310	0.762000
C	-1.674951	2.640982	1.113068
H	-2.116801	2.338577	2.073369
H	-1.273459	3.660710	1.175263
H	-2.421689	2.578003	0.316223
Sn	-0.942562	-0.240218	-0.163188
Cl	-0.518803	0.934446	-2.202939
Cl	-3.295862	0.063311	-0.310661
Cl	-0.671974	-2.404192	-1.103884
Cl	-1.062860	-1.041624	2.098264
H	0.287340	1.227053	2.552172
H	1.747416	-1.655777	-0.830522
O	3.165073	-1.214381	0.582366
C	2.655833	-2.199434	1.463885
H	3.446501	-2.426424	2.187860
H	1.762013	-1.832760	1.992951
H	2.389701	-3.116099	0.911828

II-β-gt

C	2.287200	0.799100	-0.139700
C	1.732800	-1.537500	-0.398900
C	3.080000	-1.966500	0.160400
C	4.136400	-0.894900	-0.077600
C	3.632000	0.439300	0.457400
H	2.970400	-2.154200	1.240800
H	3.362900	-2.917400	-0.314900
H	5.080900	-1.173300	0.409800
H	4.346400	-0.810700	-1.157100
H	3.509400	0.407600	1.551400
H	4.328100	1.257900	0.221500

H	1.782300	-1.419300	-1.495800
O	1.334200	-0.260100	0.149300
C	0.650000	-2.523100	-0.050300
H	0.853000	-3.472300	-0.566600
H	0.605300	-2.700500	1.036400
O	-0.597500	-2.008600	-0.501600
H	2.342800	0.883900	-1.242600
C	-1.647300	-2.982400	-0.433900
H	-1.313500	-3.888500	-0.956800
H	-1.879900	-3.209800	0.617100
H	-2.521800	-2.559100	-0.932200
Sn	-0.923600	0.191700	0.041000
Cl	-0.857500	-0.650500	2.268800
Cl	-3.242000	-0.169000	-0.393900
Cl	-1.257700	2.449100	0.695600
Cl	-0.343300	0.612100	-2.237100
O	1.743200	1.921400	0.421200
C	1.835200	3.098500	-0.356700
H	1.332100	3.891000	0.207400
H	2.886700	3.383800	-0.524300
H	1.326500	2.969700	-1.326400

II-α-gt

C	-2.262800	-0.741800	0.445000
C	-1.623100	1.397600	-0.597900
C	-2.876000	2.055300	-0.030300
C	-3.983000	1.051600	0.264700
C	-3.422100	-0.059500	1.142200
H	-2.605500	2.578600	0.901400
H	-3.208200	2.818500	-0.749800
H	-4.821500	1.555200	0.765300
H	-4.369600	0.619600	-0.670700
H	-3.067300	0.340600	2.104500
H	-4.175600	-0.830900	1.355800
H	-1.790000	1.025400	-1.620400
O	-1.212500	0.257000	0.200300
C	-0.483000	2.391900	-0.573200
H	-0.675900	3.174200	-1.320900
H	-0.382600	2.852500	0.422500
O	0.734400	1.730800	-0.902900
H	-1.769000	-1.477600	1.094200
C	1.840300	2.618700	-1.111000
H	1.558000	3.351400	-1.878100
H	2.097400	3.123700	-0.168300
H	2.680900	2.011800	-1.458900
Sn	0.969800	-0.232800	0.117700
Cl	1.028900	1.089200	2.114000
Cl	3.332300	-0.255400	-0.154000
Cl	0.666300	-2.306400	1.238200
Cl	0.545500	-1.036500	-2.089200
O	-2.671700	-1.284400	-0.745200
C	-2.320800	-2.633200	-0.991900
H	-2.850100	-3.307000	-0.298200
H	-2.633600	-2.861000	-2.017700
H	-1.236700	-2.792300	-0.906000

III-β

C	-0.009668	-1.913232	-0.982620
C	-1.987670	-1.279394	0.280152
C	-2.029491	-2.645721	0.943200
C	-1.425993	-3.703320	0.020492
C	-0.009843	-3.304802	-0.394262
H	-1.469926	-2.600160	1.891497
H	-2.062209	-3.819583	-0.872817
H	0.657771	-3.282548	0.479804
H	-2.580542	-1.287560	-0.648946
O	-0.622920	-0.981489	-0.105640
C	-2.478334	-0.159465	1.170451
H	-3.485157	-0.417947	1.527879
H	-1.807674	-0.032771	2.031518
O	-2.488004	1.086724	0.500975
Sn	1.083707	0.502551	0.181716
Cl	1.529032	-0.942778	2.029146
Cl	0.281595	2.237966	1.559109
Cl	3.331549	1.057060	-0.281676
Cl	0.174816	1.410425	-1.838781
C	-3.479727	1.330289	-0.370500
O	-4.355515	0.533694	-0.600127
C	-3.328870	2.686264	-0.988819
H	-3.306637	3.452200	-0.201416
H	-2.365262	2.729283	-1.516260
H	-4.154989	2.875714	-1.681993
H	-3.075404	-2.888253	1.180326
H	-1.401494	-4.680408	0.521540
H	0.405549	-4.006394	-1.131786
H	-0.501239	-1.856379	-1.970878
O	1.287913	-1.357223	-1.062145
C	1.993780	-1.500742	-2.300891
H	2.971497	-1.033071	-2.148767
H	2.119040	-2.568088	-2.521134
H	1.445536	-0.989934	-3.104651

III-ts

C	3.105593	-0.433375	0.365130
C	0.978020	1.899400	-0.526442
C	1.831289	1.718233	-1.783547
C	3.262237	1.248927	-1.519575
C	3.470527	-0.215815	-1.037274
H	1.312662	1.022982	-2.461968
H	1.905188	2.684441	-2.308291
H	3.823843	1.309786	-2.462175
H	3.766170	1.935318	-0.819465
H	2.832060	-0.887410	-1.631595
H	4.527682	-0.484498	-1.167692
H	1.584726	2.460470	0.214158
O	0.640383	0.688004	0.072868
C	-0.251523	2.760560	-0.788785
H	0.031353	3.746807	-1.174083
H	-0.949808	2.275603	-1.484077
O	-0.925856	3.034207	0.457099
H	2.057163	-0.569022	0.657288
Sn	-0.825825	-0.617335	-0.157679
Cl	-1.245752	-0.097243	-2.495366
Cl	-2.855562	-1.885838	-0.030230
Cl	0.659538	-2.450284	-0.712617
Cl	-0.481216	-0.913085	2.251412
O	3.987726	-0.410688	1.255475
C	3.625096	-0.608670	2.646902
H	4.166973	-1.498085	2.982988
H	3.971221	0.280113	3.183310
H	2.537929	-0.737201	2.730846
C	-1.780591	2.183988	0.945512
O	-2.071003	1.112970	0.402501
C	-2.401873	2.589938	2.235346

H	-2.149143	3.619162	2.507816
H	-3.488940	2.454389	2.167841
H	-2.029624	1.888508	2.997523

III-α

C	2.012077	-0.734468	1.006466
C	2.058168	0.622992	-0.986187
C	2.730262	-0.472095	-1.802291
C	3.534138	-1.392646	-0.884390
C	2.668939	-1.886535	0.272356
H	1.955838	-1.040535	-2.338881
H	4.404175	-0.847436	-0.486150
H	1.864631	-2.539482	-0.095960
H	2.823545	1.184545	-0.430001
O	1.202136	0.036603	0.036115
C	1.300424	1.614719	-1.835887
H	2.001131	2.053877	-2.554062
H	0.460537	1.162232	-2.375460
O	0.831456	2.724801	-1.048919
H	1.270919	-1.069818	1.747042
Sn	-1.086429	-0.397644	0.016413
Cl	-0.799118	-1.058522	-2.259741
Cl	-3.451176	-0.324183	-0.081826
Cl	-0.771237	-2.617733	0.834502
Cl	-0.925955	0.583726	2.197544
C	-0.345335	2.651978	-0.508494
O	-1.056272	1.643254	-0.616148
C	-0.794181	3.850409	0.240823
H	-0.072619	4.669482	0.169413
H	-1.776417	4.157467	-0.143112
H	-0.934323	3.541366	1.287468
H	3.385054	-0.005145	-2.553157
H	3.922501	-2.248492	-1.454058
H	3.258039	-2.462843	0.999708
O	2.965862	0.068017	1.565660
C	2.516786	1.036730	2.491298
H	1.942181	0.568445	3.306130
H	3.409696	1.522248	2.902107
H	1.880723	1.792494	2.004342

IV-β

C	1.732437	-1.921328	0.414897
C	2.108908	0.460949	0.476635
C	3.102193	0.394924	-0.690603
C	3.711768	-0.999539	-0.791835
C	2.622412	-2.068416	-0.797370
H	2.558016	0.636282	-1.615934
H	3.874635	1.160614	-0.535178
H	4.321592	-1.069773	-1.702919
H	4.391691	-1.169148	0.059836
H	1.986489	-1.987777	-1.692180
H	3.056476	-3.077810	-0.774457
H	2.635721	0.379581	1.443501
O	1.128875	-0.564112	0.392570
O	0.188870	1.860517	0.150063
H	2.319847	-1.938459	1.354244
Sn	-1.054359	0.091873	-0.154568
Cl	-0.130475	0.038354	-2.358504
Cl	-2.816242	1.600832	-0.616297
Cl	-2.199653	-1.967446	-0.372975
Cl	-1.218978	0.182860	2.231338
O	0.741036	-2.834858	0.418828
C	0.204852	-3.175772	1.687673
H	-0.571705	-3.927676	1.511500
H	0.989129	-3.602437	2.334076
H	-0.247245	-2.300488	2.176622
C	1.378085	1.775012	0.455610
O	2.106108	2.798584	0.731747
C	1.503223	4.106153	0.677942
H	2.297621	4.804210	0.952472
H	1.142000	4.302156	-0.338638
H	0.672234	4.155912	1.391535

IV-ts

C	-3.216296	0.431080	0.085018
C	-0.108593	1.886029	-0.755295
C	-0.863948	2.810722	0.218733
C	-2.385918	2.812052	0.079144
C	-3.159972	1.690296	0.824052
H	-0.589677	2.537775	1.249388
H	-0.502721	3.835000	0.044035
H	-2.758842	3.745085	0.523156
H	-2.685836	2.843507	-0.980220
H	-2.620374	1.452366	1.758888
H	-4.181208	2.014286	1.062146
H	-0.206819	2.297118	-1.779398
O	-0.520184	0.570958	-0.724064
C	1.370794	1.957981	-0.409874
O	1.966300	0.969898	0.022049
H	-2.284106	-0.094810	-0.172818
Sn	0.672306	-0.835074	0.053136
Cl	-0.100179	-0.069876	2.257070
Cl	2.431113	-2.045017	1.123688
Cl	-1.042719	-2.517219	-0.099492
Cl	1.578595	-1.272624	-2.143558
O	-4.322150	-0.024161	-0.284826
C	-4.414489	-1.263876	-1.035146
H	-3.422569	-1.725083	-1.112499
H	-5.114470	-1.898905	-0.483990
H	-4.824132	-0.997614	-2.014540
O	1.942523	3.106943	-0.565832
C	3.330625	3.229717	-0.216948
H	3.598130	4.267735	-0.431329
H	3.467714	3.004550	0.847866
H	3.928720	2.540080	-0.825248

IV-α

C	1.980505	-1.500866	-0.070661
C	1.826539	0.912260	0.241824
C	2.732502	1.173549	-0.965712
C	3.650548	-0.020969	-1.207900
C	2.828439	-1.302355	-1.308059
H	2.092279	1.341153	-1.845695
H	3.304437	2.092287	-0.776844
H	4.222468	0.137200	-2.132511
H	4.376897	-0.109089	-0.385438
H	2.148159	-1.259025	-2.171631
H	3.469496	-2.187041	-1.423845
H	2.413668	0.882681	1.173896
O	1.115431	-0.315979	0.107859
O	-0.410543	1.788491	0.181544
H	1.249391	-2.315218	-0.183618
Sn	-1.139958	-0.237810	-0.106428
Cl	-0.574467	0.109137	-2.409227

Cl	-3.364027	0.523259	-0.307810
Cl	-1.308790	-2.598846	-0.296648
Cl	-1.112162	-0.248271	2.289085
O	2.768006	-1.652496	1.030750
C	2.110749	-2.142397	2.186661
H	1.665317	-3.131665	1.990330
H	2.868742	-2.234860	2.972663
H	1.317102	-1.453907	2.513717
C	0.790127	1.996294	0.368534
O	1.264136	3.158448	0.643820
C	0.354004	4.271696	0.739258
H	-0.383209	4.075772	1.526837
H	0.975734	5.134249	0.990486
H	-0.148694	4.417252	-0.224227

V-β

C	-2.123564	1.360828	0.291414
C	-1.971800	-1.023261	0.642478
C	-2.972629	-1.313438	-0.484595
C	-3.873273	-0.104706	-0.716491
C	-3.043002	1.163617	-0.891065
H	-2.402466	-1.539563	-1.398049
H	-3.557544	-2.203275	-0.214007
H	-4.499244	-0.277448	-1.602564
H	-4.559206	0.016132	0.138748
H	-2.418864	1.112311	-1.796249
H	-3.685839	2.051739	-0.967168
H	-2.490990	-0.944244	1.614197
O	-1.241192	0.170361	0.404977
O	0.202881	-2.004731	0.421789
H	-2.685793	1.371538	1.245894
Sn	1.033470	-0.044027	-0.129471
Cl	0.101261	-0.464302	-2.287787
Cl	3.074549	-1.190101	-0.460009
Cl	1.706619	2.168229	-0.623000
Cl	1.214335	0.188339	2.243629
O	-1.350189	2.455337	0.144062
C	-0.897039	3.071252	1.339946
H	-0.298397	3.938504	1.041852
H	-1.753404	3.406949	1.947100
H	-0.268416	2.385490	1.926536
C	-0.973455	-2.141485	0.744336
O	-1.460848	-3.267465	1.152559
H	-0.774944	-3.958434	1.155862

V-ts

C	-3.092127	-0.503673	0.103579
C	-0.648724	1.896033	-0.788576
C	-1.675210	2.531664	0.169903
C	-3.107943	2.017973	0.029547
C	-3.467172	0.721974	0.805340
H	-1.330695	2.384513	1.205108
H	-1.678921	3.614687	-0.023067
H	-3.776909	2.783034	0.446545
H	-3.389767	1.918733	-1.030633
H	-2.888746	0.703178	1.747132
H	-4.539686	0.690024	1.035824
H	-0.875405	2.233063	-1.819801
O	-0.596064	0.520952	-0.732031
C	0.717250	2.468411	-0.450914
O	1.612058	1.750380	-0.010113
H	-2.037025	-0.690181	-0.149606
Sn	1.001319	-0.397566	0.048304
Cl	0.021152	0.095416	2.244849
Cl	3.071186	-0.929936	1.113113
Cl	-0.040352	-2.561531	-0.076642
Cl	1.989127	-0.513047	-2.152609
O	-3.978070	-1.318609	-0.239048
C	-3.646063	-2.541349	-0.948677
H	-2.556706	-2.640158	-1.025950
H	-4.086846	-3.356630	-0.367299
H	-4.124787	-2.463957	-1.929797
O	0.871377	3.746353	-0.625596
H	1.766133	4.017867	-0.358625

V-α

C	2.026719	-1.214510	-0.384692
C	1.807830	1.013819	0.573644
C	2.690589	1.626967	-0.519192
C	3.634265	0.571510	-1.089570
C	2.847358	-0.660210	-1.528598
H	2.035019	2.015593	-1.313312
H	3.244187	2.471291	-0.086000
H	4.183583	0.994837	-1.941617
H	4.378832	0.285090	-0.331172
H	2.151212	-0.406310	-2.341852
H	3.512404	-1.458043	-1.886626
H	2.411082	0.743175	1.455944
O	1.127809	-0.148640	0.109746
O	-0.449527	1.798171	0.797896
H	1.318349	-1.992894	-0.705837
Sn	-1.131733	-0.077048	-0.100134
Cl	-0.575061	0.977117	-2.178085
Cl	-3.371022	0.662828	-0.073924
Cl	-1.231443	-2.265684	-1.011863
Cl	-1.102523	-0.814049	2.175093
O	2.835613	-1.626669	0.630434
C	2.212888	-2.424499	1.622411
H	1.784608	-3.336555	1.174964
H	2.988949	-2.703397	2.344059
H	1.411663	-1.868920	2.132568
C	0.746725	1.984507	1.011567
O	1.194189	3.045973	1.596458
H	0.464668	3.648757	1.825137

VI-β

C	-2.519202	0.414809	0.152341
C	-1.536644	-1.562435	1.082509
C	-2.159143	-2.445500	0.010382
C	-3.449959	-1.821837	-0.509682
C	-3.211009	-0.378750	-0.947367
H	-1.440977	-2.559297	-0.815842
H	-2.347855	-3.441342	0.438323
H	-3.848900	-2.408753	-1.349036
H	-4.213924	-1.842757	0.285928
H	-2.576548	-0.351195	-1.846868
H	-4.160985	0.118891	-1.188262
H	-2.239413	-1.440797	1.925409
O	-1.277457	-0.243360	0.546927
C	-0.229329	-2.075312	1.647425
H	-0.306972	-3.144601	1.881241
O	0.800802	-1.869374	0.678883
H	-3.136652	0.435922	1.066285
C	1.348361	-3.038252	0.054948

H	1.843046	-3.649078	0.821613
H	0.553372	-3.608519	-0.443613
H	2.086976	-2.698561	-0.676880
Sn	0.969729	0.200003	-0.110281
Cl	0.214081	-0.694989	-2.183165
Cl	3.276989	-0.331301	-0.379428
Cl	1.151143	2.436531	-0.885410
Cl	1.102470	0.845126	2.186421
H	0.057754	-1.529446	2.553565
C	-1.866042	2.901414	1.196291
H	-1.582738	3.934322	0.956167
H	-2.756142	2.908753	1.840107
H	-1.025843	2.412519	1.705483
S	-2.218702	2.091225	-0.382513

VI-ts

C	-3.098272	-0.038723	0.585831
C	-0.646609	1.913919	-0.474141
C	-1.677727	2.897390	0.061092
C	-3.129867	2.430216	-0.006561
C	-3.574991	1.306044	0.972529
H	-1.409177	3.155952	1.098684
H	-1.609355	3.825657	-0.529573
H	-3.776633	3.285703	0.235119
H	-3.385890	2.131008	-1.035951
H	-3.179400	1.525906	1.975704
H	-4.673983	1.314457	1.014508
H	-0.967512	1.566143	-1.477946
O	-0.516064	0.828406	0.394792
C	0.718546	2.572514	-0.635220
H	0.720748	3.315419	-1.447609
H	1.037093	3.050473	0.307816
O	1.635821	1.528770	-0.961639
H	-2.083751	-0.343047	0.874046
C	2.985922	1.953289	-1.117876
H	3.568563	1.071987	-1.405160
H	3.036239	2.713443	-1.910227
H	3.366683	2.360818	-0.169206
Sn	1.028138	-0.369250	0.133421
Cl	2.168095	0.656457	2.014764
Cl	3.063468	-1.458234	-0.536114
Cl	0.004497	-2.123209	1.414441
Cl	0.058180	-1.056691	-2.016641
C	-2.983691	-2.496154	-0.602537
H	-2.741144	-2.959026	0.361032
H	-3.587329	-3.172740	-1.218577
H	-2.069318	-2.189968	-1.130776
S	-4.010796	-1.036847	-0.329782

VI-α

C	2.204969	-0.351143	-0.616379
C	1.450043	1.336932	0.990217
C	1.900979	2.469016	0.081646
C	3.084937	2.022487	-0.773963
C	2.740840	0.741806	-1.531439
H	1.054912	2.772620	-0.553792
H	2.176616	3.327388	0.711998
H	3.357108	2.812625	-1.487783
H	3.965542	1.860064	-0.130291
H	1.953490	0.951675	-2.270642
H	3.609609	0.346978	-2.076818
H	2.272188	1.033383	1.657117
O	1.110863	0.169728	0.197158
C	0.245637	1.646179	1.850333
H	0.344380	2.659506	2.263157
O	-0.916216	1.599334	1.017777
C	-2.078936	2.213652	1.589281
H	-2.397655	1.655876	2.481783
H	-1.830935	3.250950	1.848557
H	-2.864158	2.192130	0.828093
Sn	-1.047090	-0.258618	-0.200814
Cl	-0.823806	1.231937	-2.054160
Cl	-3.420559	-0.234483	-0.293532
Cl	-0.526012	-2.225568	-1.425087
Cl	-1.016098	-1.366638	1.929387
H	0.129616	0.925396	2.672187
H	1.743291	-1.158108	-1.196470
C	2.559318	-2.466446	1.114533
H	3.212260	-2.957215	1.847704
H	1.649973	-2.110428	1.616495
H	2.292468	-3.183410	0.326314
S	3.490746	-1.072820	0.430993

References

1. Xue, J. L.; Cecioni, S.; He, L.; Vidal, S.; Praly, J.-P. *Carbohydr. Res.*, **2009**, *344*, 1646
2. Gaussian 16, Revision C.01, Frisch, M. J.; Trucks, G. W.; Schlegel, H. B.; Scuseria, G. E.; Robb, M. A.; Cheeseman, J. R.; Scalmani, G.; Barone, V.; Petersson, G. A.; Nakatsuji, H.; Li, X.; Caricato, M.; Marenich, A. V.; Bloino, J.; Janesko, B. G.; Gomperts, R.; Mennucci, B.; Hratchian, H. P.; Ortiz, J. V.; Izmaylov, A. F.; Sonnenberg, J. L.; Williams-Young, D.; Ding, F.; Lipparini, F.; Egidi, F.; Goings, J.; Peng, B.; Petrone, A.; Henderson, T.; Ranasinghe, D.; Zakrzewski, V. G.; Gao, J.; Rega, N.; Zheng, G.; Liang, W.; Hada, M.; Ehara, M.; Toyota, K.; Fukuda, R.; Hasegawa, J.; Ishida, M.; Nakajima, T.; Honda, Y.; Kitao, O.; Nakai, H.; Vreven, T.; Throssell, K.; Montgomery, J. A., Jr.; Peralta, J. E.; Ogliaro, F.; Bearpark, M. J.; Heyd, J. J.; Brothers, E. N.; Kudin, K. N.; Staroverov, V. N.; Keith, T. A.; Kobayashi, R.; Normand, J.; Raghavachari, K.; Rendell, A. P.; Burant, J. C.; Iyengar, S. S.; Tomasi, J.; Cossi, M.; Millam, J. M.; Klene, M.; Adamo, C.; Cammi, R.; Ochterski, J. W.; Martin, R. L.; Morokuma, K.; Farkas, O.; Foresman, J. B.; Fox, D. J. Gaussian, Inc., Wallingford CT, 2016.
3. Chai, J.-D.; Head-Gordon, M. *Phys. Chem. Chem. Phys.* **2008**, *10*, 6615.
4. (a) Weigend, F.; Ahlrichs, R. *Phys. Chem. Chem. Phys.* **2005**, *7*, 3297-3305; (b) Weigend, F. *Phys. Chem. Chem. Phys.* **2006**, *8*, 1057.
5. Hay, P. J.; Wadt, W. R. *J. Chem. Phys.* **1985**, *82*, 299.
6. Scalmani, G.; Frisch, M. J. *J. Chem. Phys.* **2010**, *132*, 114110.
7. Ribeiro, R. F.; Marenich, A. V.; Cramer, C. J. &; Truhlar, D. G. *J. Phys. Chem. B* **2011**, *115*, 14556.
8. (a) Gonzalez, C.; Schlegel, H. B. *J. Chem. Phys.* **1989**, *90*, 2154–2161; (b) Gonzalez, C.; Schlegel, H. B. *J. Phys. Chem.* **1990**, *94*, 5523.
9. Rappoporta, D.; Furche, F. *J. Chem. Phys.*, **2010**, *133*, 134105
10. (a) Keith, T. A.; Bader, R. F. W. *Chem. Phys. Lett.*,**1992**, *194*, 1-8; (b) Keith, T. A.; Bader, R. F. W. *Chem. Phys. Lett.*,**1993**, *210*, 223.
11. (a) Becke, A. D. *J. Chem. Phys.* **1993**, *98*, 5648; (b) Lee, C.; Yang, W.; Parr, R. G. *Phys. Rev. B*, **1988**, *37*, 785; (c) Vosko, S. H.; Wilk, L.; Nusair, M. *Can. J. Phys.* **1980**, *58*, 1200; (d) Stephens, P. J.; Devlin, F. J.; Chabalowski, C. F.; Frisch, M. J. *Phys. Chem.* **1994**, *98*, 11623.
12. Marenich, A. V.; Cramer, C. J.; Truhlar, D. G. *J. Phys. Chem. B*, **2009**, *113*, 6378-6396.
13. (a) Khan, A. A.; Kalisch, T.; Espinosa Ferao, A.; Streubel, R. *Dalton Trans.* **2023**, *52*, 3275. (b) Hersh, W. H.; Chan, T.-Y., *Beilstein J. Org. Chem.* **2023**, *19*, 36.
14. Gao, P.; Zhang, J.; Chen, H. *Int. J. Quant. Chem.* **2021**, *121*, e26482.
15. (a) Bursch, M.; Gasevic, T.; Stückrath, J. B.; Grimme, S. *Inorg. Chem.* **2021**, *60*, 272. (b) Ludwig, M.; Franz, D.; Espinosa Ferao, A.; Bolte, M.; Hanusch, F.; Inoue, S. N. *Nat. Chem.* **2023**, *15*, 1452.
16. Stückrath, J. B.; Gasevic, T.; Bursch, M.; Grimme, S. *Inorg. Chem.* **2022**, *61*, 3903.
17. (a) Unione, L.; A. Ardá, A.; J. Jiménez-Barbero, Millet, O. *Curr. Opin. Struct. Biol.* **2021**, *68*, 9; (b) A. Gimeno, A.; Valverde, P.; Ardá, A. Jiménez-Barbero, J. *Curr. Opin. Struct. Biol.* **2020**, *62*, 22; (c) Valverde, P.; Quintana, J. I.; Santos, J. I.; Ardá, A.; Jiménez-Barbero, J. *ACS Omega*, **2019**, *4*, 13618.
18. Martins, J. C.; Biesemans, M.; Willem, R. *Progr. Nucl. Magn. Res. Spectrosc.* **2000**, *36*, 271.
19. Wolzak, L. A.; Hermans, J. J.; de Vries, F.; van den Berg, K. J.; Reek, J. N. H.; Tromp, M.; Korstanje, T. J. *Catal. Sci. Technol.* **2021**, *11*, 3326.
20. Otera, J. *J. Organomet. Chem.* **1981**, *221*, 57.
21. Hunter, B. K.; Reeves. L. W. *Can. J. Chem.* **1968**, *46*, 1399.
22. (a) van den Berghe, E. V.; van der Kelen. G. P. *J. Organometal. Chem.* **1968**, *11*, 479; (b) J. D. Kennedy, J. D.; McFarlane. W. *J. Chem. Soc. Perkin Trans. 2*, **1974**, 146; (c) Fratiello, A.; Peak, S.; Schuster, R. E.; Davis, D. *J. Phys. Chem.* **1970**, *74*, 3730.
23. (a) Smith, P. J.; White, R. F. M.; Smith, L. *J. Organomet. Chem.* **1972**, *40*, 341; (b) Tupčiauskas, A. P.; Sergeyev, N. M.; Ustynyuk, Y. A. *Org. Magn. Res.* **1971**, *3*, 655; (c) Chapman, A. C.; Davies, A. G.; Harrison, P. G.; McFarlane, W. *J. Chem. Soc. C*, **1970**, 821.

24. Kennedy, J. D.; McFarlane, W.; Smith, P. J.; White, R. F. M.; Smith, L. *J. Chem. Soc. Perkin Trans. 2*, **1973**, 1785.

25. (a) Davies, A. G.; Harrison, P. G.; Kennedy, J. D.; Mitchell, T. N.; Puddephatt, R. J.; McFarlane, W. *J. Chem. Soc. C*, **1969**, 1136; (b) Gutowsky, H. S.; Juan, C. *J. Chem. Phys.* **1962**, *37*, 2198.

26. Smith, P. J.; Smith. L. *Inorg. Chim. Acta Reviews*, **1973**, *7*, 11.

27. Holecek, J.; Nadvornik; N.; Handlir, K. *J. Organomet. Chem.* **1983**, *241*, 177.

28. Davies, A. G.; Harrison, P. G.; Kennedy, J. D.; Mitchell, T. N.; Puddephatt, R. J.; McFarlane. W. *J. Chem. Soc. C*, **1969**, 1136.

29. Gutowsky, H. S.; Juan, C., *J. Chem. Phys.* **1962**, *37*, 2198.

30. (a) van den Berghe, E. V.; van der Kelen, G. P. *J. Organomet. Chem.*, **1971**, *26*, 207-213; (b) Kennedy, J. D., *J. Chem. Soc., Perkin Trans. 2*, **1977**, 242; (c) van den Berghe, E. V.; van der Kelen, G. P., *J. Mol. Struct.*, **1974**, *20*, 147; (d) Kennedy, J. D.; McFarlane, W. *J. Chem. Soc., Dalton Trans.*, **1973**, 2134.

31. Smith, P. J.; Tupčiauskas, A. P., *Annu. Rep. NMR Spectrosc.*, **1978**, *8*, 291.
